# Data-driven machine learning optimisation of silver nanoparticle synthesis

**DOI:** 10.1039/d6na00557h

**Published:** 2026-09-21

**Authors:** Joseph Carver, Y. M. John Chew, Semali Perera, Sourav Chatterjee

**Affiliations:** a Department of Chemical Engineering, University of Bath Bath BA2 7AY UK jc2899@bath.ac.uk sc999@bath.ac.uk

## Abstract

Precise control of silver nanoparticle (Ag-NP) size is critical for application-specific performance, yet identification of optimal synthesis conditions remains reliant on trial-and-error experimentation. Here, we report a machine learning inverse design framework trained on a combined experimental dataset spanning batch and continuous flow reactor configurations. Five regression algorithms were evaluated as candidate surrogate models, with Gaussian process (GP) regression emerging as the best-performing model (coefficient of determination (*R*^2^) = 0.91 and cross-validation score = 0.80). A random sampling strategy was employed to explore the parameter space and identify synthesis conditions capable of achieving user-defined target Ag-NP hydrodynamic diameters. To differentiate between the multiple feasible solutions, a composite candidate scoring function was implemented to rank synthesis conditions according to the error, estimated Ag-NP dispersity, and posterior uncertainty. The framework was validated across 21 inverse design predictions spanning a target size range of 20–70 nm, achieving average size errors of 3.4% and 7.4% for the batch and flow reactors, respectively. Collectively, this work establishes an experimentally validated inverse design framework capable of identifying synthesis conditions for target Ag-NP hydrodynamic diameters *via* citrate- and tannic acid-mediated reduction of silver nitrate.

## Abstract 2

Surrogate-based inverse design framework that identifies the synthesis conditions required to reach a user-defined target Ag-NP size.

## Graphical abstract

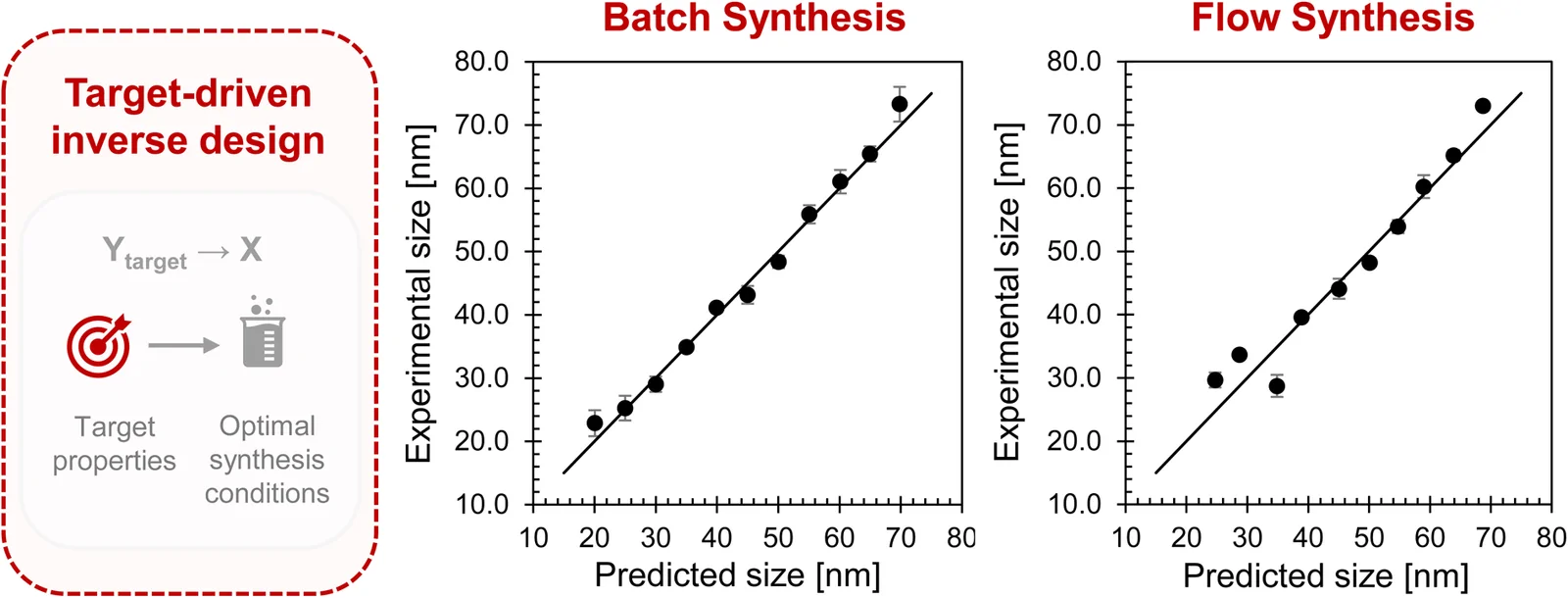

## 1 Introduction

Silver nanoparticles (Ag-NPs) are of growing importance across a wide range of biomedical and technological applications, including catalysis, energy storage and surface-enhanced Raman scattering detection.^1–5^ Their size- and shape-dependent properties enable precise tuning of physicochemical characteristics for targeted applications.^6,7^ Consequently, their performance is highly sensitive to synthesis conditions, which strongly governs the particle size, morphology and colloidal stability. However, despite increasing demand for precise and reproducible nanoparticle synthesis, exploration and optimization of Ag-NP synthesis spaces remain reliant on heuristic approaches, resulting in significant experimental cost, time expenditure and limited reproducibility.^8^

Chemical reduction of silver salts remains the most widely employed route for the synthesis of Ag-NPs due to its simplicity, low cost, short reaction time and high yields.^9,10^ In this method, reducing agents such as sodium citrate, ascorbate, sodium borohydride and tannic acid reduce silver ions (Ag^+^) to metallic silver (Ag^0^), while stabilising agents adsorb onto particle surfaces to inhibit growth and aggregation.^11–15^ The choice and concentration of these reagents, together with temperature and reactor configuration, strongly influence the rates of nucleation and growth, thereby providing effective means to tailor Ag-NP size and morphology.^16–18^ However, these parameters are interdependent and influence nucleation and growth simultaneously, thus making precise, reproducible and predictable control over Ag-NP size a significant challenge.

To address the challenges of navigating complex, nonlinear synthesis spaces efficiently, machine learning (ML) has emerged as a powerful tool for data-driven optimisation and process control in NP synthesis.^19–23^ In Ag-NP synthesis, Nathanael *et al.* (2023) employed extreme gradient boosting (XGBoost) and random forest (RF) regression models to predict Ag-NP sizes in the range of 5–20 nm, synthesised in a microfluidic reactor under varying kinetic and hydrodynamic conditions, achieving a coefficient of determination (*R*^2^) of 0.66.^24^ Furxhi *et al.* (2024) applied regression models, including linear regression, RF, XGBoost and support vector regression, on existing data collected from 219 studies, with RF and XGBoost achieving the highest predictive performance for core size prediction, both attaining *R*^2^ values of 0.70.^25^ Most recently, Richard *et al.* (2026) utilised a convolutional neural network framework to enable data-efficient prediction of morphological and optical properties of Ag nanoprisms from synthesis parameters and *in situ* time-resolved UV-visible spectroscopy.^20^

While these frameworks focus on forward prediction, such ML methodologies have advanced considerably for Au-NP synthesis, where data-driven modelling frameworks have been employed for both optimisation and inverse design of morphological and optical properties. Kim *et al.* (2026) integrated a droplet-flow microreactor with *in situ* small- and wide-angle X-ray scattering characterisation and Gaussian process-guided active learning, achieving control over Au-NP size (4–60 nm) and polydispersity.^26^ Further, Vaddi *et al.* (2025) demonstrated inverse design of Au-NPs through a differentiable modelling framework, enabling retrosynthesis of target spectral properties *via* gradient-based optimisation.^27^ These demonstrations, however, depend on automated synthesis platforms coupled to *in situ* or in-line characterisation, and their translation to Ag-NP synthesis under conventional laboratory conditions remains largely unexplored.

In materials science and manufacturing, surrogate-based inverse design frameworks have enabled efficient exploration of high-dimensional parameter spaces; from the design of photonic meta-surfaces to the optimisation of additive manufacturing.^28–30^ These frameworks couple a trained surrogate model with an optimisation algorithm to identify input conditions satisfying a desired target output. Mahdian *et al.* (2026) benchmarked seven optimisation strategies for surrogate-based inverse design of electrospun nanofiber diameter, finding comparable performance between particle swarm optimisation (PSO) and Bayesian optimisation (BO) when coupled with an XGBoost model.^31^ Within Ag-NP synthesis, Mekki-Berrada *et al.* (2021) demonstrated BO with deep neural networks to optimise the synthesis of Ag nanoprisms with targeted optical properties in a droplet flow reactor, while Shafaei *et al.* (2020) utilised a hybrid artificial neural network PSO approach to minimise Ag-NP size.^32,33^ More recently, Nathanael *et al.* (2025) employed a degree-3 polynomial surrogate model coupled with Latin hypercube sampling for the inverse design of target Ag-NP sizes in a microfluidic reactor, representing an early demonstration of data-driven inverse modelling in Ag-NP flow synthesis.^34^ However, in complex synthesis systems, multiple combinations of process parameters can result in the same target output, and a limitation of many optimisation-based approaches is their convergence to a single solution without means of differentiating between the multiple feasible candidates that may satisfy the target quantity.^35^

This study focuses on the development of a framework for the inverse modelling of quasi-spherical Ag-NP hydrodynamic diameter *via* the reduction of silver nitrate using sodium citrate (SC) and tannic acid (TA) (Fig. 1). Existing demonstrations of inverse design in Ag-NP synthesis return a single optimised condition rather than a ranked set of feasible alternatives, and the closest analogous work in Au-NP synthesis depends on automated platforms coupled to *in situ* or in-line characterisation. In this work, an experimentally validated surrogate-based inverse design framework has been developed which instead enumerates a large population of candidate conditions and differentiates between the multiple feasible solutions, ranking them according to predicted size error, dispersity and posterior uncertainty. The surrogate is trained on a single dataset pooled across batch and continuous flow reactors, with reactor configuration included as an explicit feature, and the framework is assessed by synthesising and characterising 21 conditions generated by the model. The limited availability of Ag-NP synthesis data in the literature, necessitated the generation of our own Ag-NP database.^25^ This work establishes a target-oriented inverse design workflow for Ag-NP synthesis that operates from a scarce, unstructured dataset generated on conventional laboratory equipment.

**Fig. 1:**
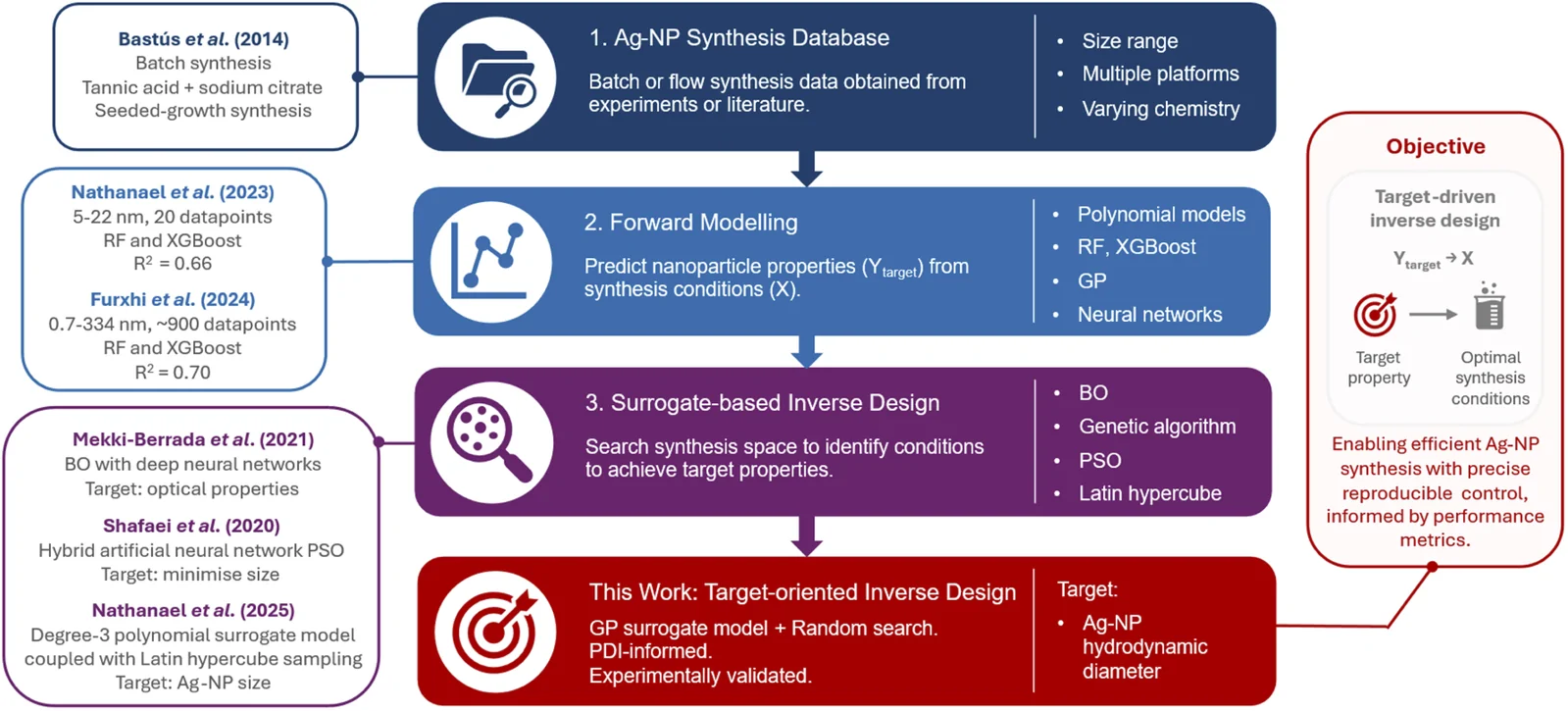
Workflow for the development of a surrogate-based, target-oriented inverse design framework for Ag-NP synthesis.

## 2 Method and materials

### 2.1 Chemicals

Silver nitrate (AgNO_3_, 99%), tannic acid (C_76_H_52_O_46_, 99%) and tri-sodium citrate dihydrate (C_6_H_5_Na_3_O_7_·2H_2_O, 98%) obtained from Sigma Aldrich was used for Ag-NP synthesis. All the chemical reagents were used as received without further purification or processing. Solutions were prepared in ultrapure water (18.2 MΩ cm) from PURELAB Chorus water purification system.

### 2.2 Synthesis of Ag-NPs

#### 2.2.1 Batch synthesis

The synthesis protocol for Ag-NPs was based on the reduction of AgNO_3_ using SC and TA.^11^ A 50 mL volume of aqueous solution containing SC (0.1–3.96 mM) and TA (0.01–0.62 mM) was prepared in a 100 mL beaker and heated using a heating mantle for 15 min under stirring at 400 rpm. Once the temperature of the mixture reached the set point (60–100 °C), 0.5 mL of AgNO_3_ (25 mM or 50 mM) was injected to initiate the reaction. The resulting colloidal Ag-NP solution was used directly for characterisation without further purification.

#### 2.2.2 Flow synthesis

Ag-NPs were synthesised in a continuous gas–liquid flow system in which the aqueous reagents comprised the dispersed phase, while nitrogen gas served as the continuous phase. The Ag-NP synthesis was translated from laboratory-scale batch to flow synthesis using the equivalent final reagent concentrations.

Fig. 2 shows the schematic of the flow reactor system. Aqueous reagents were pre-heated and fed into the microreactor at 80 µL min^−1^ using 2.5 ± 0.05 mL Hamilton syringes at a 1 : 1 volumetric ratio. The aqueous streams and nitrogen gas were introduced to the microreactor *via* a quad mixer to generate the segmented flow. A nitrogen gas flow rate of 1.1 mLn min^−1^ was chosen to ensure stable droplet formation resulting in a residence time of approximately 12 min. The microfluidic tubing was submerged within a silicon oil bath heated *via* a heating mantle. Upon exiting the reactor, the colloidal Ag-NP solution was collected in a glass vial. A 2.7 bar(g) pressure was maintained in the reactor using a back pressure regulator to avoid boiling and bubble formation within the microreactor.^36^

**Fig. 2:**
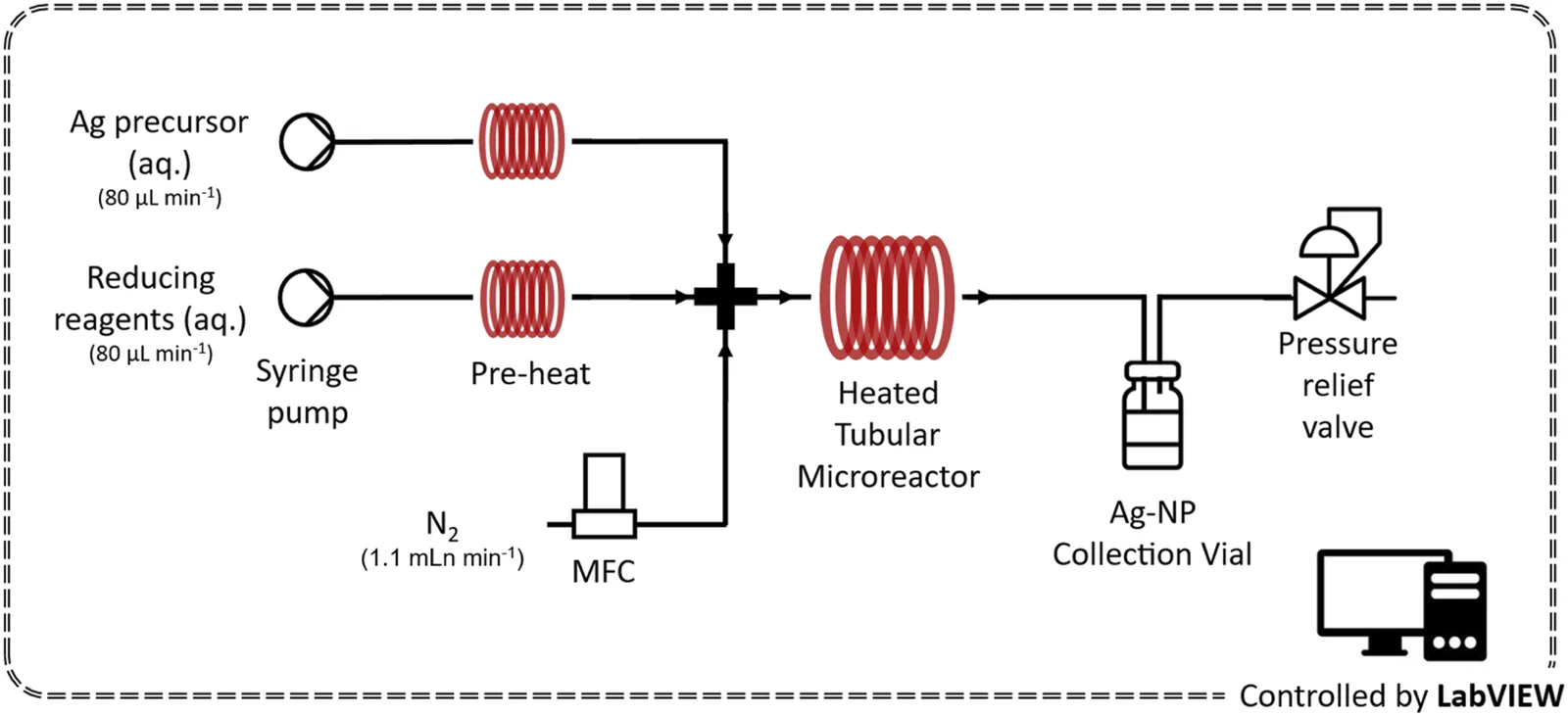
Schematic of the flow reactor system for the continuous synthesis of Ag-NPs.

### 2.3 Advanced nanoparticle characterisation

#### 2.3.1 Dynamic light scattering (DLS)

The hydrodynamic diameter (*Z*-average) and particle size distribution (PSD) of the colloidal Ag-NP product were measured using a Malvern Zetasizer Ultra-ZSU3305 nanoparticle analyser at 25 °C with a fixed scattering angle of 173°.

#### 2.3.2 UV-visible spectroscopy

The UV-visible spectra of the Ag-NPs were acquired using an Agilent Cary 100 spectrophotometer with 3 mL plastic cuvettes over a wavelength range of 300–800 nm. To monitor the evolution of the Ag-NP surface plasmon resonance (SPR) during batch synthesis, 0.20 mL aliquots were collected at 1 min intervals, diluted with 1.5 mL UPW, and analysed by UV-vis spectroscopy.

#### 2.3.3 Transmission electron microscopy (TEM)

The Ag-NPs were visualised using Jeol 1010 TEM. Ten microliters of each solution were drop-casted onto a carbon coated grid and left to dry for 24 h. Average TEM particle size and PSD of the samples were measured using ImageJ software.

### 2.4 Machine learning framework

#### 2.4.1 Software libraries

The ML analysis was performed using Python programming language. All models were developed using standard scientific computing libraries, including *NumPy* and *pandas* for data handling, and *scikit-learn* for implementation of RF, XGBoost, and Gaussian process (GP) regression models. Data visualisation was conducted using Matplotlib. For the GP model, numerical features were standardised using the *StandardScaler* from the Python *scikit-learn* package.

#### 2.4.2 Feature selection and analysis

For each experiment, the hydrodynamic diameter was recorded. Each dataset was divided into five inputs features and one predicted output, and their investigated ranges are summarised in Table 1. Features were selected based on their established influence on Ag-NP size and their practical tunability as direct synthesis inputs.^1,16^ The boundaries of the parameter space were constrained to conditions that provide monodisperse PSD, avoiding secondary nucleation and/or agglomeration. Correlation analysis of the input features was performed using the Pearson correlation coefficient.^24^ To obtain lists of feature importance for each algorithm after training, the permutation method available in *scikit-learn* was utilised.

**Table 1:** Input parameters used to predict hydrodynamic diameter and their investigated ranges

Feature	Range investigated
Temperature (°C)	60–125^a^
Concentration of AgNO_3_ in the reaction mixture = [Ag]_rxn_ (mM)	0.10–0.50
Concentration of TA in the reaction mixture = [TA]_rxn_ (mM)	0.01–0.62
Concentration of SC in the reaction mixture = [SC]_rxn_ (mM)	0.10–3.96
Reactor configuration (—)	1 (flow) or 0 (batch)

a The upper temperature range (100–125 °C) is accessible only in the pressurised flow reactor.

#### 2.4.3 Training and testing

A total of five regression algorithms were evaluated: first- and second order polynomial multiple linear regression (MLR), RF, XGBoost, and GP. The data was divided into an 80% training set and a 20% held-out test set using a random split with a fixed random seed of 42 to ensure reproducibility. To optimise model performance, hyperparameter tuning was conducted using randomised search with *k*-fold cross-validation. The training dataset was divided into *k* = 5 folds. For each iteration, four folds were used for model training, and the remaining fold was used for validation. This process was repeated until each fold had served once as the validation set. *RandomizedSearchCV* was used to sample random combinations of hyperparameters from predefined parameter distributions for each algorithm. The average cross-validation (CV) score across the five folds was calculated for each hyperparameter combination and the set producing the best mean performance was selected as the optimal model configuration. The final model was then evaluated on the held-out test dataset that was not used during training or hyperparameter optimisation.

#### 2.4.4 Evaluation metrics

The performance of the developed models was described through the root mean square error (RMSE), the mean absolute error (MAE), the mean absolute percentage error (MAPE), and the *R*^2^ value which were calculated through eqn (1)–(4), respectively.^32^1
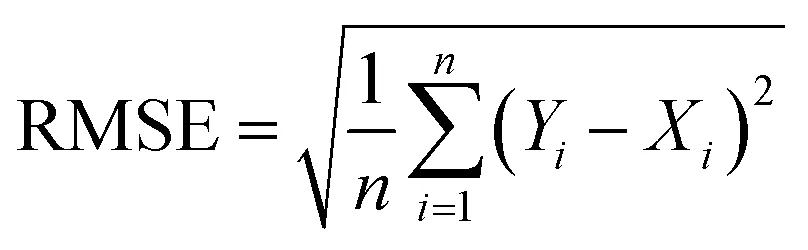
2
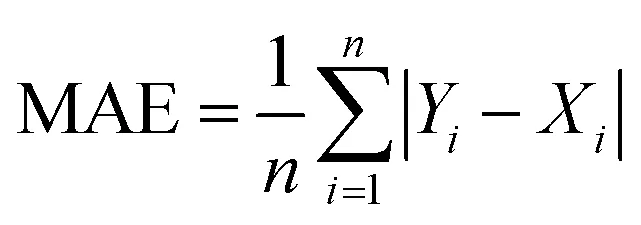
3
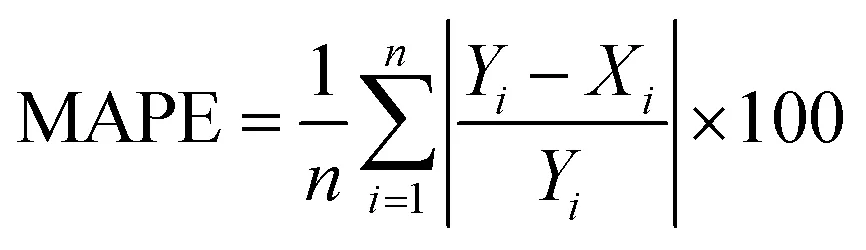
4
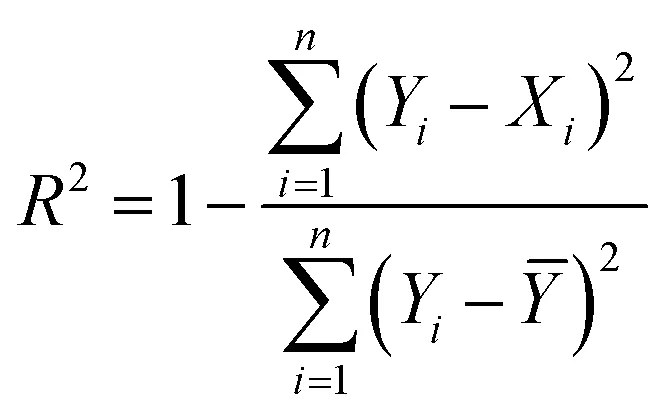
Where *n* is the number of data within training and testing datasets, *X*_*i*_ is the predicted *i*th value, *Y*_*i*_ is the actual *i*th value, and *Ȳ* is the mean of the true values. The closer the value of *R*^2^ is to 1 and the values of RMSE, MAE and MAPE are to zero, the better the fit of the model to the data.

### 2.5 Optimisation

#### 2.5.1 Surrogate-based inverse design

The objective of this framework was to identify combinations of the input features that produce the target Ag-NP size. Inverse design was performed by stochastic sampling of the synthesis parameter space (Table S2). A total of 500 000 candidate parameter sets were generated from the search space. This population size was chosen to ensure high coverage of the chemical space whilst remaining computationally efficient. For each candidate, the trained surrogate model was used to obtain a predicted diameter *f*_surrogate_(*x*) and posterior uncertainty *σ*(*x*). Candidate conditions satisfying eqn (5) are retained.

5 |*f*_surrogate_(*x*) − *d*_target_| ≤ *δ*

where *x* is the vector representing a set of process parameters within the feasible domain, *d*_target_ is the target diameter, and *δ* = 2 nm is the size tolerance. This tolerance was chosen to represent an acceptable error in the prediction and the measurement uncertainty of DLS.

#### 2.5.2 Polydispersity index estimation

For each feasible candidate, an empirical polydispersity index (PDI) estimate was obtained from the single nearest neighbour in a size-conditioned subset of the training dataset. The nearest neighbour within this subset was identified by *k*-nearest-neighbour (*k*NN) lookup, and its measured PDI value was assigned as the PDI estimate for the candidate.

#### 2.5.3 Evaluator function

Feasible candidates were subsequently ranked by a composite score combining normalised error, normalised empirical PDI (PDI(*x*)), and normalised posterior uncertainty (*σ*(*x*)) normalised by maximum values of 2.0 nm, 0.265, and 28.6 nm, respectively.6
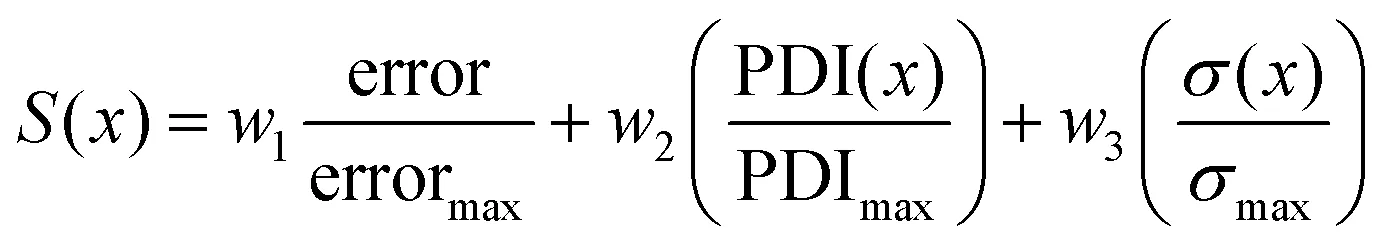


The candidate with the lowest composite score was selected as the generated procedure, representing the conditions predicted to produce the target NP size with the lowest PDI neighbourhood and highest model confidence among the feasible candidates.

### 2.6 Ag-NP kinetics

The synthesis of Ag-NPs was modelled by three steps: reduction of Ag^+^ ions to Ag atoms; the formation of Ag nuclei; and subsequent growth of these nuclei to generate Ag-NPs. The Finke–Watzky (F–W) two-step of slow, continuous nucleation and fast autocatalytic surface growth has frequently been applied to the chemical synthesis of Ag-NPs.^24,37^Eqn (7) and (8) correspond to the pseudo elementary steps of slow nucleation and fast autocatalytic growth.7
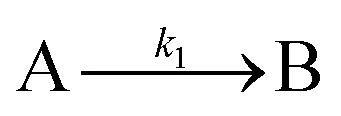
8
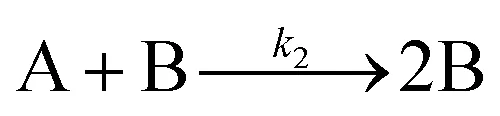
where, *k*_1_ and *k*_2_ are the nucleation and growth rates, and [A] and [B] are the concentration of the silver precursor (AgNO_3_) and Ag-NPs/nuclei, respectively. Eqn (9) corresponds to the overall reaction rate.9
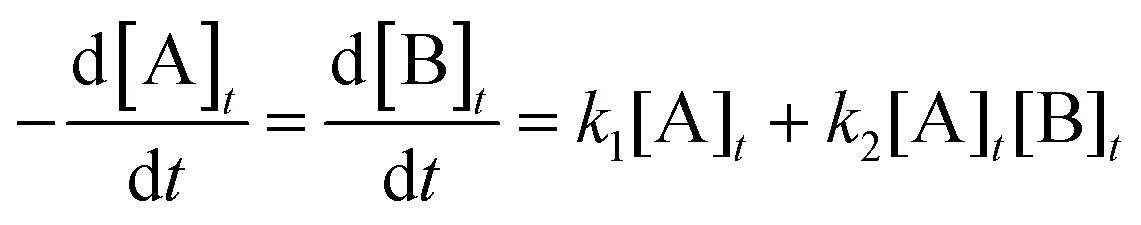


Eqn (10) represents the integrated form of the F–W mechanism which was used to fit the experimental kinetic datasets.10
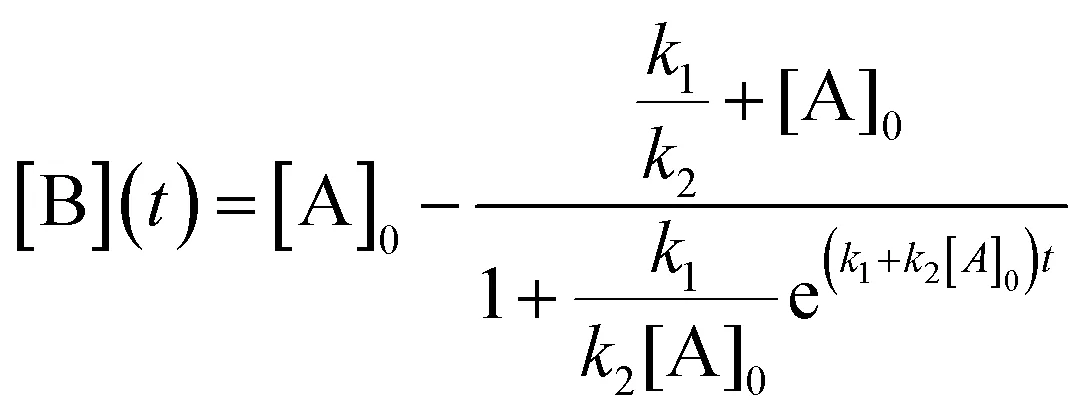


The nucleation (*k*_1_) and growth (*k*_2_) constants were estimated using the linearised form shown in eqn (11) under the assumption that nucleation happens more slowly than growth.11
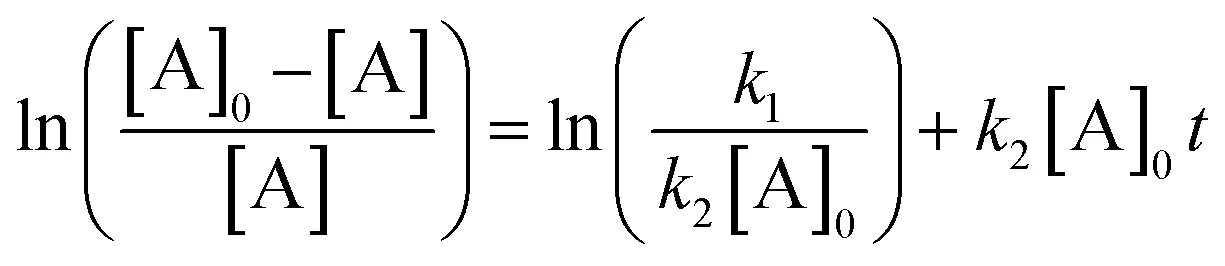


## 3 Results

### 3.1 Generating and characterising NP library

Ag-NPs were synthesised with cumulant hydrodynamic diameters between 22 and 70 nm (Fig. 3(a)). The data consists of observational data from unstructured experimental exploration driven by chemical intuition, previous results and practical constraints. The dataset was divided into quartile-based intervals: Q1 (20–32 nm), Q2 (32–44 nm), Q3 (44–56 nm), and Q4 (56–70 nm). The resulting distribution is skewed towards Q1, Q2, and Q3, which contain 27, 43, and 32 datapoints, respectively. Comparatively, Q4 is moderately underrepresented with 17 datapoints. The non-uniform distribution of observations of the predicted output (*Z*-average) reflects the complexity of the chemical space.

**Fig. 3:**
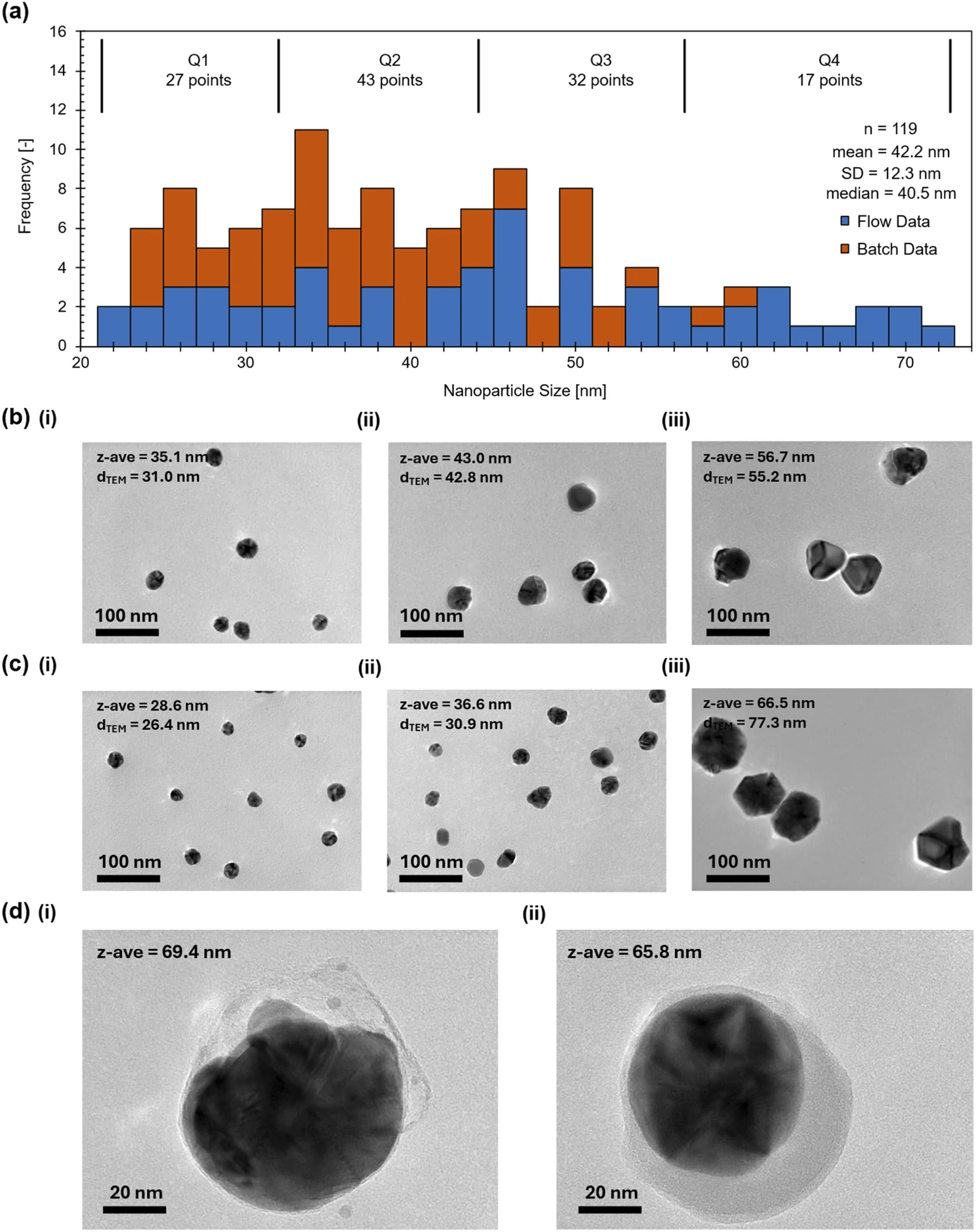
(a) Histogram of the Ag-NP size distribution for flow and batch datasets. (b) TEM micrographs for Ag-NPs synthesised in the batch reactor in the intervals (i) and (ii) Q2, and (iii) Q4. (c) TEM micrographs for Ag-NPs synthesised in the flow reactor in the intervals (i) Q1, (ii) Q2, and (iii) Q4. (d) TEM micrographs for Ag-NPs in Q4 under (i) high [TA]_rxn_ and (ii) low [SC]_rxn_.

TEM micrographs of Ag-NPs synthesised using the batch and flow reactor are presented in Fig. 3(b) and (c), respectively, along with their measured DLS hydrodynamic diameter and TEM average diameters. TEM images of the Ag-NPs synthesised in the intervals Q1 and Q2 (Fig. 3(b)(i)–(ii) and 3(c)(i)–(ii)) reveal quasi-spherical morphologies. Whereas Ag-NPs within the interval Q4 (Fig. 3(b)(iii) and 3(c)(iii)) exhibited a mixture of quasi-spherical, non-spherical, and faceted morphologies depending on the synthesis conditions. The disparity between *Z*-average and TEM-measured diameter shown in Fig. 3(c)(iii) occurs from the inherent assumption in DLS that particles are perfectly spherical when calculating hydrodynamic radius; for non-spherical particles, the *Z*-average therefore represents an equivalent sphere diameter rather than a true physical dimension.^38^ Additional TEM micrographs are shown in SI S.1.

The *Z*-average hydrodynamic diameter alone is insufficient to fully characterise the NP population, as equivalent hydrodynamic sizes can correspond to different distributions. Therefore, the *Z*-average was reported along with the measured PDI to assess the degree of size non-uniformity within each sample. When selecting optimal synthesis conditions, it is critical to consider both target hydrodynamic diameter and dispersity, as both govern the properties and performance of the NPs in downstream applications. This effect was illustrated by comparing large Ag-NPs (Q4) synthesised in the flow reactor at elevated temperatures (>100 °C) under either high [TA]_rxn_ (>0.40 mM) or low [SC]_rxn_ (<0.25 mM). Representative TEM images of Ag-NPs synthesised under these two conditions are given in Fig. 3(d)(i) and (ii), respectively. The NPs synthesised under high [TA]_rxn_ exhibit irregular morphologies and a broader size distribution (PDI = 0.15), whereas NPs synthesised under low [SC]_rxn_ exhibit faceted and quasi-spherical morphologies with a narrower distribution (PDI = 0.12).

To facilitate the generation of a sufficiently large dataset necessary for ML modelling, batch and flow reactor datasets were combined into a single dataset. Including both reactors enables a wider chemical space to be explored, leveraging the benefits of each configuration, such as longer reaction times in the batch reactor and higher temperatures in the flow reactor. To standardise the thermal history of both reactor configurations and minimise differences in heat transfer, reagents underwent thermal preconditioning at the reaction temperature prior to mixing. Consequently, the primary difference between the reactors was confined to the hydrodynamics of the reaction. To capture for these platform specific differences, reactor configuration was encoded as a binary feature. To assess the influence of reactor configuration on Ag-NP hydrodynamic diameter, equivalent synthesis conditions, shown in Table 2, were evaluated across both platforms. For conditions tested within quartiles Q2, Q3, and Q4 the reactors yielded Ag-NPs with hydrodynamic diameters within 2–4 nm of each other. The close agreement between platforms under this synthesis condition is attributable to the reaction operating in the kinetically controlled regime, wherein the reduction of AgNO_3_ by SC and TA occurs on a longer timescale than the mixing in either reactor, ensuring reagents are homogeneous prior to the induction period. However, for conditions that yield Ag-NPs within Q1, the reduction of AgNO_3_ by SC and TA occurs on a timescale comparable to the mixing, resulting in reactor specific differences in the hydrodynamic diameter.

**Table 2:** Comparison between Ag-NPs synthesised within the batch and flow reactor under equivalent synthesis conditions

Temp. (°C)	[Ag]_rxn_ (mM)	[TA]_rxn_ (mM)	[SC]_rxn_ (mM)	Batch hydrodynamic diameter (nm)	Flow hydrodynamic diameter (nm)
90	0.50	0.03	3.96	17.5	24.3
70	0.49	0.30	0.98	34.0	36.6
90	0.50	0.10	0.48	43.0	46.3
90	0.50	0.19	0.46	58.6	56.4

The dataset comprised 58 data points generated on the flow reactor and 61 on the laboratory-scale batch reactor, yielding a total of 119 data points. The dataset was divided into a training set of 95 datapoints and a held-out test set of 24 data points, with equal representation from each reactor configuration. Given the size of the database, more sophisticated algorithms such as neural networks were unsuitable. The algorithms chosen for this study were first and second order MLR, RF, XGBoost, and GP.

### 3.2 Correlation analysis

The correlation matrix reveals instances of weak to moderate correlations between variables (Fig. 4). Although these correlations describe relationships within the dataset, they do not indicate causal effects, since all variables were manually set prior to synthesis. Most of the correlations therefore reflect the design and constraints of the experimental conditions rather than intrinsic chemical dependencies. For example, temperature exhibits a strong correlation with reactor configuration, a consequence of the flow reactor enabling controlled synthesis at elevated temperatures (>100 °C).

**Fig. 4:**
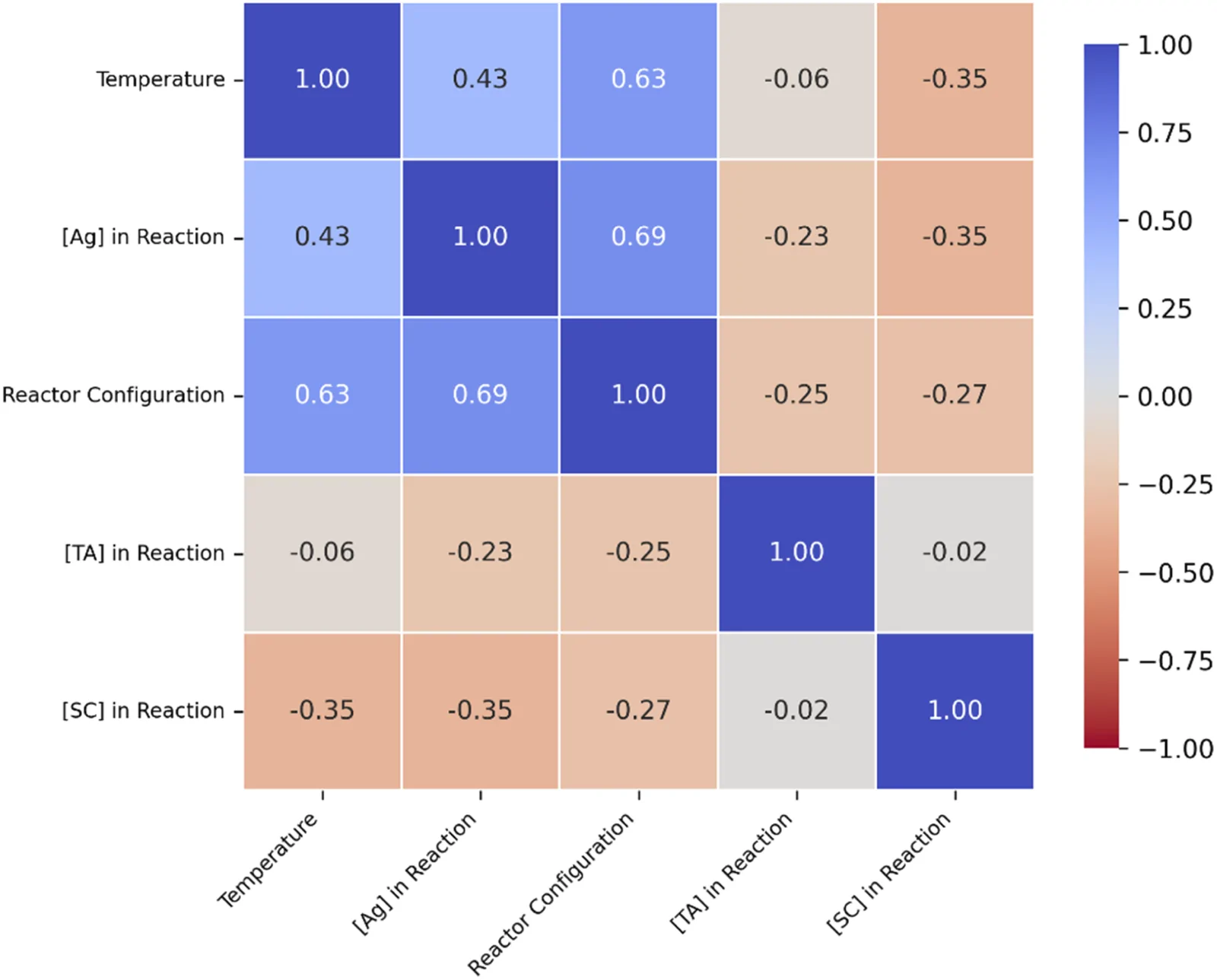
The correlation matrix of all variables. Blue indicates a positive correlation while red indicates a negative correlation.

A limitation of the flow reactor was the fixed residence time, governed by the length of the reactor tubing. As a result, conditions within the flow reactor were limited to synthesis reactions that would complete within the 12 min residence time. This was confirmed by monitoring the change in SPR by UV-vis spectroscopy and the colour change of the reaction mixture. It was assumed that, within the chemical space explored by the flow reactor, the Ag precursor was consumed within the fixed residence time, enabling meaningful comparison between both the reactor configurations. Conditions that required an extended residence time were synthesised using the batch reactor. The negative correlation between [SC]_rxn_ and reactor configuration, therefore, reflects the design and constraints of the experimental system, as lower [SC]_rxn_ results in slower nucleation and growth rates.

Feature importance analysis using the permutation method revealed that [SC]_rxn_ and [TA]_rxn_ are the dominant factors governing Ag-NP size across all regression models (Fig. S4), reflecting the coupled influence of both reagents on nucleation and growth dynamics. [Ag]_rxn_ showed low permutation importance; while less directly influential on size, it serves primarily to normalise the ratios of reducing and stabilising agents across experiments. The reactor configuration showed the lowest permutation importance, consistent with the reactors operating in largely non-overlapping regions of the chemical space. The strong correlation between reactor configuration and both temperature (*r* = 0.63) and [Ag]_rxn_ (*r* = 0.69) in the correlation matrix suggests that the influence of the reactor configuration is likely confounded with these variables.

### 3.3 Regression model performance

The statistical characteristics of the regression models was evaluated on the test dataset across the five metrics: CV score, RSME, MAE, MAPE, and the standard deviation of the residuals (Table 3). Among the evaluated methods, the GP model demonstrated the best overall predictive performance across all metrics. It exhibited the lowest RMSE (3.87 nm), MAE (3.35 nm), MAPE (8.43%), and standard deviation of residuals (3.85 nm), indicating both high accuracy and consistency. The XGBoost model showed the second-best performance, with RMSE (4.16 nm), MAE (3.46 nm), MAPE (8.88%), and standard deviation of residuals (3.92 nm), also reflecting string predictive capability. The second-order polynomial MLR achieved comparable performance to the RF model, showing that simple polynomial models can be used to predict NP as effectively as complex models and suggesting that a sizeable portion of the synthesis space variance is governed by quadratic feature interactions. The second order polynomial MLR underpredicts the hydrodynamic diameter for elevated temperatures (>100 °C) and low [SC]_rxn_ (∼0.1 mM) conditions, suggesting a higher order polynomial relationship in this region of the chemical space and justifying the use of more complex ML algorithms.

**Table 3:** Prediction results of the trained models for the held-out test set for 1st and 2nd order MLR, RF, XGBoost, GP trained on the combined batch and flow dataset and GP models trained on the batch and flow only datasets

Model	*R* ^2^	CV score	RMSE (nm)	MAE (nm)	MAPE (%)	Std. deviation of residuals (nm)
1st order MLR	0.61	0.48	8.29	6.43	16.06	8.29
2nd order MLR	0.83	0.49	5.51	4.24	10.43	5.52
RF	0.87	0.71	4.55	4.00	10.70	4.39
XGBoost	0.90	0.79	4.16	3.46	8.88	3.92
GP (combined)	0.91	0.80	3.87	3.35	8.43	3.85
GP (batch only)	0.37	0.78	6.78	4.76	16.00	5.34
GP (flow only)	0.89	0.43	4.34	3.47	6.95	4.25

Fig. 5 shows the centralised distribution of residuals for all the models. The median scores for the first order MLR, second order MLR, RF, XGB, and GP models are −0.47, −0.81, −2.1, −0.66, and −0.18 nm, respectively. Although all models showed negative median residuals, the magnitude of the bias was small, indicating only a slight systematic tendency toward overprediction. The first-order MLR models exhibited the largest spread of residuals, reflecting the limited ability of the linear model to capture the nonlinear relationships present within the synthesis space. The RF, XGBoost, and GP models experienced the comparable spread of residuals, indicating improved predictive consistency across the dataset over the MLR models.

**Fig. 5:**
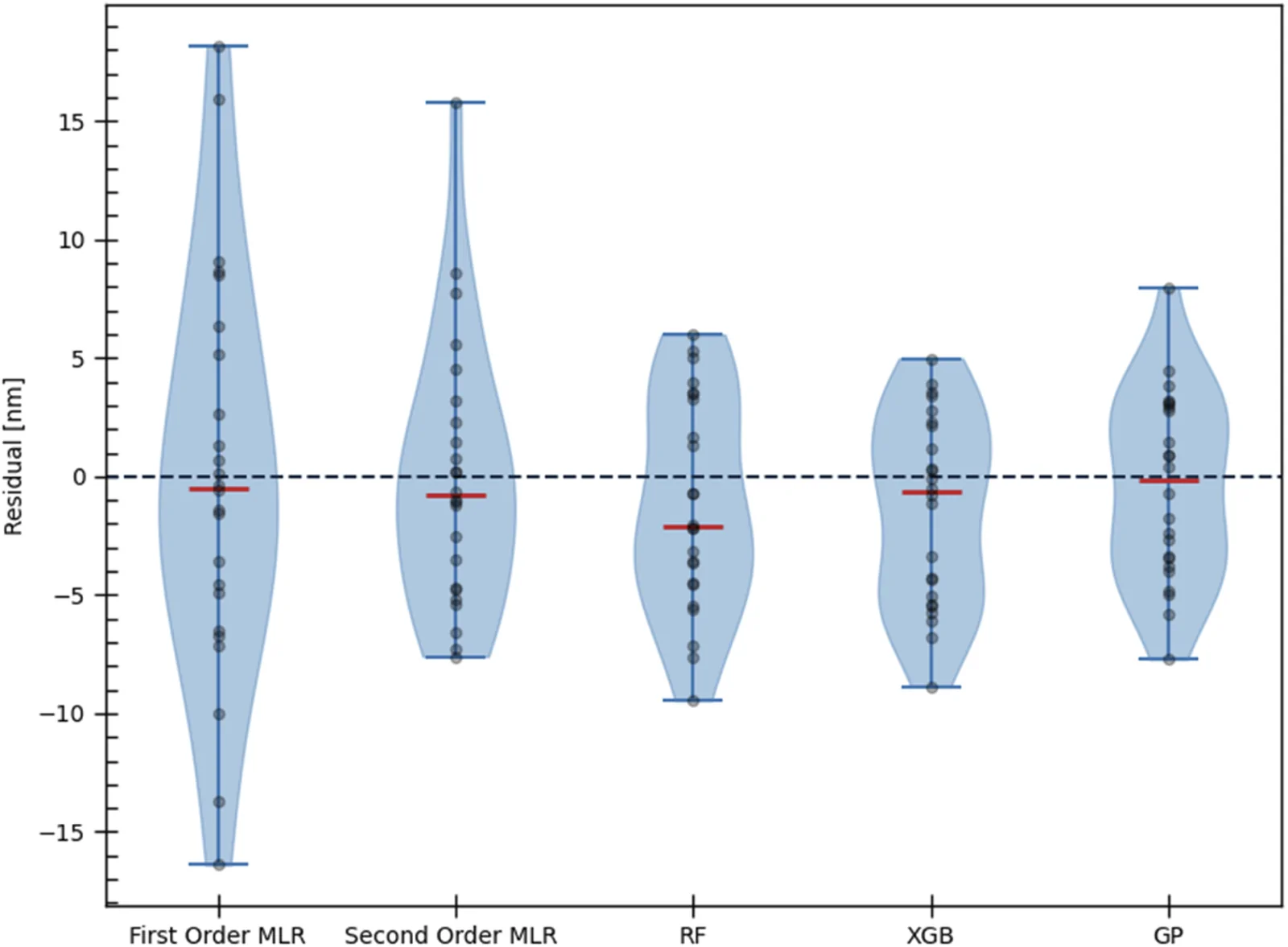
Violin plot of the distribution of the residuals of the test set. The dashed line shows the ideal value of zero for residuals, the red lines and show the median residual, respectively. Circles denote individual residuals.

Evaluation of the regression models revealed that the GP model exhibited the best predictive performance. GP models are particularly well-suited for surrogate-based optimisation due to their probabilistic nature, providing mean predictions alongside uncertainty estimates. This enables informed navigation of the chemical space, balancing exploration of uncertain regions with exploitation of known high-performing conditions.

To validate the combined datasets from the batch and flow reactors, separate GP models were trained on the batch and flow datasets individually using the same train-test splits (Table 3). The combined dataset GP model outperformed the individually trained models, consistent with its wider chemical space coverage and greater number of training points improving generalisation across the synthesis space.

### 3.4 Kinetic rationalisation

The global feature–response relationships captured by the GP surrogate model can be rationalised through the underlying reaction kinetics of Ag-NP formation, providing insight into the synthesis trends learned by the trained forward model. Fig. 6(a) presents a representative fit of the F–W model applied to the chemical synthesis of Ag-NPs, whereby the time dependence of the concentration of Ag in the nanoparticles/nuclei were collected by following the change in SPR by UV-vis spectroscopy during batch synthesis. The corresponding rate constants for nucleation (*k*_1_) and growth (*k*_2_), and the sigmoidal parameters under varying [TA]_rxn_ and [SC]_rxn_ under constant conditions (temperature = 70 °C, [TA]_rxn_ = 0.04 mM, [SC]_rxn_ = 1 mM, and [Ag]_rxn_ = 0.49 mM) are summarised in Table 4. The ratio of *k*_2_[A]/*k*_1_ represents the relative importance of autocatalytic growth *versus* nucleation.

**Fig. 6:**
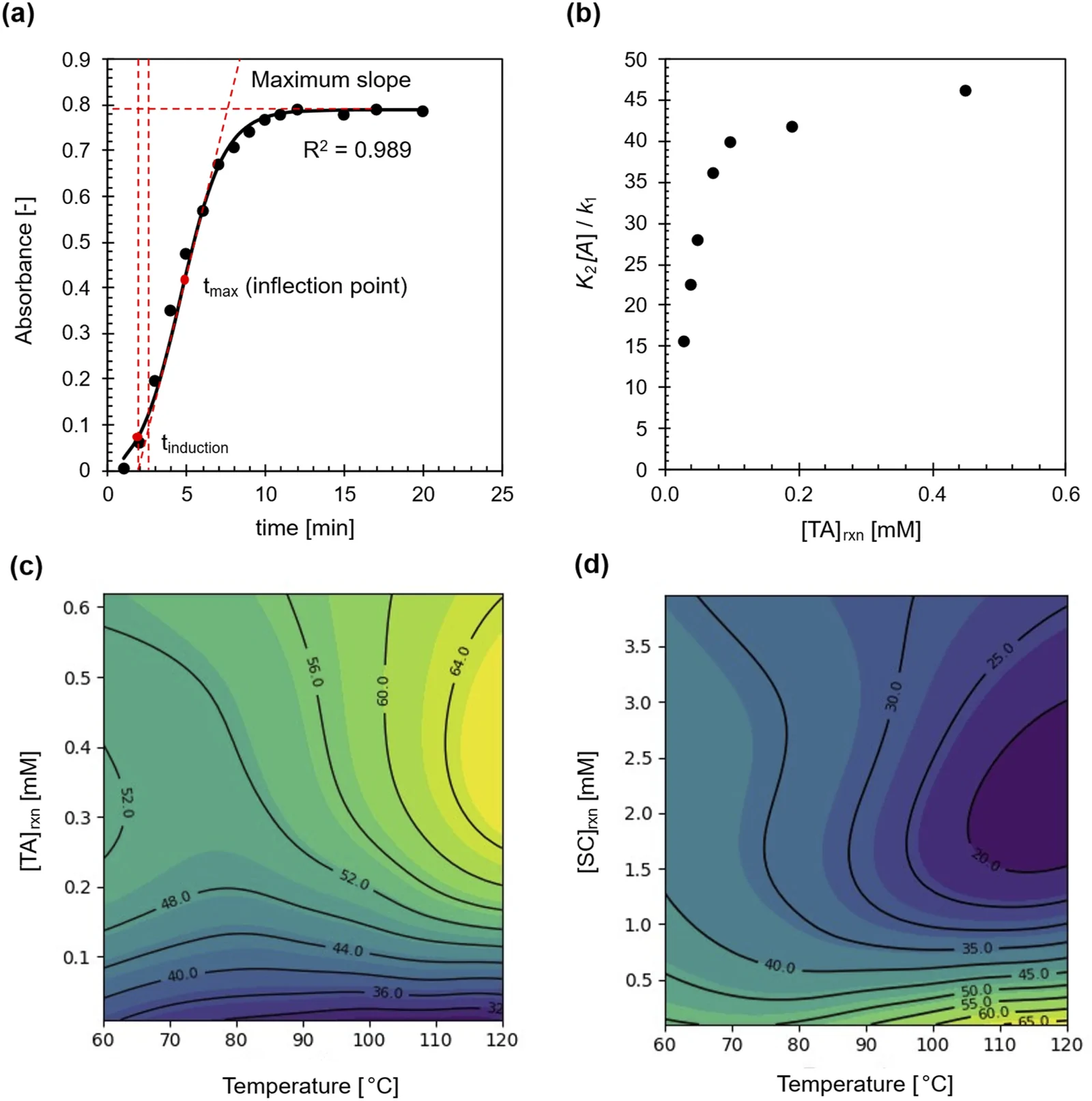
(a) Sigmoidal-shaped kinetic curve for the formation of Ag-NPs obtained by measuring the change in SPR by UV-vis spectroscopy and illustration of sigmoidal parameters: “induction period” *t*_induction_, “inflection point” *t*_max_, and maximum rate ([TA]_rxn_ = 0.05). Curve-fit to the F–W two step model (*R*^2^ = 0.989) adapted from Bentea *et al.* (2017).^39^ (b) Ratio of rate constants extracted from kinetic curves at varying [TA]_rxn_. PDPs showing predicted Ag-NP size as a function of reagent concentration and temperature, showing the effects of (c) [TA]_rxn_ and (d) [SC]_rxn_.

**Table 4:** Kinetic constants and sigmoidal parameters for varying [TA]_rxn_ and [SC]_rxn_

	*k*_1_ (min^−1^)	*k*_2_[A] (min^−1^)	*t*_induction_ (min)	*t*_max_ (min)	Maximum rate (min^−1^)	DLS size (nm)
**[TA]**_**rxn**_ **(mM)**						
0.02	—	—	—	—	—	28.0
0.03	0.036	0.56	1.5	4.6	0.124	31.2
0.04	0.025	0.70	1.6	5.4	0.117	36.3
0.05	0.025	0.58	1.6	4.6	0.149	37.1
0.07	0.020	0.72	1.4	4.9	0.149	37.1
0.10	0.019	0.76	1.8	4.7	0.167	41.2
0.20	0.016	0.67	2.3	5.5	0.136	46.6
0.45	0.013	0.58	2.3	6.4	0.010	49.2

**[SC]**_**rxn**_ **(mM)**						
0.20	0.021	0.22	3.2	9.8	0.050	44.5
0.25	0.010	0.26	4.3	12.0	0.043	46.1
0.50	0.032	0.40	2.1	5.8	0.095	39.8
0.75	0.028	0.44	2.1	5.9	0.102	38.8
1.00	0.025	0.70	1.6	5.4	0.117	36.3
1.50	0.045	0.71	1.1	3.7	0.156	35.0
2.00	0.044	0.77	1.2	3.5	0.168	33.5

The direct dependence between [TA]_rxn_ and size is due to the ability of TA to form strong [Ag_2_^+^–TA] complexes with Ag^+^, which undergo slower transformations compared to the un-complexed Ag_2_^+^.^11^Fig. 6(b) shows how the ratio *k*_2_[A]/*k*_1_ rises sharply at low [TA]_rxn_ and plateaus at increasing [TA]_rxn_, reflecting a transition from nucleation-dominated to autocatalytic growth regimes. Under low [TA]_rxn_, the reduction of Ag^+^ is slow, autocatalytic surface growth is negligible, and the nucleation term dominates. As [TA]_rxn_ increases, the formation of [Ag_2_^+^–TA] complexes increase, thereby suppressing *k*_1_. Concurrently, increased [TA]_rxn_ enhances electron transfer at particle surfaces, increasing *k*_2_[A] and promoting autocatalytic growth on existing particles.

The [SC]_rxn_ had the inverse effect on the NP size. Extracted values for *k*_1_ and *k*_2_[A]_0_ both increased with increasing [SC]_rxn_, consistent with the role of SC as both a reducing and stabilising agent. At low [SC]_rxn_ (<1 mM), there is weak surface passivation, and the autocatalytic term dominates. This corresponds to a high *k*_2_[A]/*k*_1_ ratio, favouring the formation of larger NPs. As [SC]_rxn_ increased, the values of both *k*_1_ and *k*_2_[A] increased due to the stronger reducing environment; however, the relative increase in *k*_1_ is higher, leading to a decrease in the ratio *k*_2_[A]/*k*_1_. This indicates a shift towards faster nucleation, generating a larger number of nuclei leading to a smaller particle size.

To extract interpretable structure from the trained model, partial dependence plots (PDPs) were employed to investigate the global feature–response relationships across the chemical space. The PDPs reveal how temperature modulates within the different regimes identified. Fig. 6(c) shows the PDP for [TA]_rxn_ as a function of the temperature of reaction. At low [TA]_rxn_, where autocatalytic growth is suppressed, temperature has negligible effect on NP size because nucleation dominates. At higher [TA]_rxn_ (>0.07 mM), increasing temperature accelerates surface-catalysed growth, enhancing the autocatalytic term *k*_2_[A][B] and resulting in the formation of larger particles. Fig. 6(d) shows the PDP for [SC]_rxn_ as a function of the temperature. At low [SC]_rxn_, the autocatalytic term dominates and an increase in temperature accelerates the surface-catalysed growth, resulting in the formation of larger NPs. At [SC]_rxn_ > 1 mM the temperature has a much smaller influence as the final NP size is governed by the rate of nucleation.

### 3.5 Experimental validation of the trained forward model

To assess the trained forward model, external Ag-NP synthesis data reported in literature was used to test the models' ability to generalise to conditions reported by Bastús *et al.* (2014), Cheng *et al.* (2016), and Ranoszek-Soliwoda *et al.* (2017).^11,40,41^ This validation test data was obtained using batch reactor configurations, reported in Table 5. To the authors knowledge there was no comparable data available for Ag-NPs synthesised in flow reported in literature.

**Table 5:** Prediction results of the trained GP surrogate model for the literature validation set

Source	Temp. (°C)	[Ag] (mM)	[TA]_rxn_ (mM)	[SC]_rxn_ (mM)	DLS exp. size (nm)	Predicted size (nm)
Bastús *et al.* (2014)	100	0.25	0.25	5.0	34.2	37.8
	100	0.25	1.00	5.0	43.6	45.9
	100	0.25	5.00	5.0	58.0	41.9
Cheng *et al.* (2016)	100	0.59	0.06	1.4	22.0^a^	26.5
	100	0.59	0.08	1.4	25.0^a^	29.5
	100	0.59	0.24	1.4	29.0^a^	46.1
Ranoszek-Soliwoda *et al.* (2017)	100	0.92	0.18	6.5	40.0	39.0

a TEM diameter.

Bastús *et al.* (2014) utilised a fixed [SC]_rxn_ of 5 mM, exceeding the maximum of 4 mM used within the model's training dataset, and varied the [TA]_rxn_ to control the Ag-NP size.^11^ The experimental DLS values measured by Bastús *et al.* (2014) were 34.2, 43.6, and 58.0 nm for [TA]_rxn_ of 0.25, 1, and 5 mM. Based on these input conditions the GP model predicted hydrodynamic diameters of 37.8, 45.9, and 41.9 nm, respectively. The model predictions follow the same trend as the experimental data and provides strong predictions for data within or close to the training set range. At high [TA]_rxn_ and [SC]_rxn_ the model underpredicted the size by 17.1 nm, a result of the uncertainty between the competing effects of [TA]_rxn_ and [SC]_rxn_ beyond the training range.

For data reported by Cheng *et al.* (2016) the model predicted hydrodynamic diameters of 26.5, 29.5 and 46.1 nm, compared to TEM-derived values of 22, 25, and 29 nm.^40^ The hydrodynamic diameter measured by DLS is typically larger than TEM-derived diameter, and the predictions for the first two datapoints are consistent with this expected offset.^42^ The larger discrepancy observed for the third datapoint was likely attributable to the two-step heating process employed by Cheng *et al.* (2016), whereby the reagent mixture was first preheated and mixed at 60 °C for 30 min before being heated to boiling. Lastly, the model provided a close prediction of 39.0 nm compared to the 40.0 nm size reported by Ranoszek-Soliwoda *et al.* (2017).^41^ These validation tests with external data from conditions reported by other research groups show that the model can capture trends well within the trained chemical space but struggles to generalise outside of this region. The model is also constrained to reduction of AgNO_3_*via* SC and TA and does not extend to alternative synthesis routes.

### 3.6 Optimisation with surrogate modelling

The trained forward model was subsequently utilised to develop a target-driven inverse design framework, whereby synthesis conditions capable of achieving a user-specified Ag-NP hydrodynamic diameter could be identified. The inverse design of NP synthesis is an inherently ill-posed problem: multiple conditions can result in the same target size, meaning no unique solution exists. Therefore, the optimisation challenge becomes finding the optimal solution among many candidates. In this case, finding the condition that simultaneously satisfies the target size while minimising dispersity, thereby promoting greater monodispersity assuring constant application performance.

Direct (random) sampling of the parameter space offers a practical approach for surrogate-based inverse design problems. By evaluating many candidate conditions against a trained forward model, it maintains the diversity of the feasible solutions. While gradient-based and Bayesian optimisation methods explore the parameter space more efficiently they are susceptible to convergence on local optima in non-convex landscapes and typically return a single candidate solution.^35^ Instead, random sampling generates a broad population of candidate conditions that satisfy the target size, allowing secondary criterion such as dispersity, yield, or colloidal stability to be evaluated simultaneously when selecting the optimal synthesis conditions.

Pourkamali-Anaraki *et al.* (2024) formalised a two-stage framework comprising a “learner” model that identifies candidate inputs whose predicted outputs closely match the target, and an “evaluator” that narrows the input space to provide more precise predictions.^43^ The present work employs a similar two-stage framework, generating candidates through random sampling before applying an analogous evaluation stage using a multi-criteria composite scoring function (eqn (6)). This scoring function integrates three terms: prediction error, GP posterior uncertainty, and a *k*NN-estimated PDI. The GP uncertainty term penalises predictions in regions of the parameter space where the surrogate is less reliable due to sparse training data. The *k*NN-estimated PDI introduces an empirical quality constraint, favouring candidates associated with narrow size distributions without requiring a dedicated dispersity predictive model.

The PSD-based PDI was incorporated as this empirical quality constraint because dispersity directly governs the suitability of the synthesised NPs for downstream applications. The measured PDI values across the training dataset ranged from 0.05 to 0.20; however, samples exhibiting higher dispersity (0.10–0.20) have poor signal-to-noise ratio that made developing a dedicated PDI predictive model unreliable. Instead, PDI was estimated for each feasible candidate using a *k*NN lookup (*k* = 1) applied to the training data, restricted to a subset of training samples whose measured diameter falls within the target size range. This provides an estimate of the dispersity expected for a candidate condition, informed by training data points with similar size and synthesis conditions, which is subsequently used within the scoring function to prioritise candidates with lower estimated dispersity. The use of a single neighbour was necessitated by the sparsity of the dataset, which prevented reliable averaging across multiple neighbours.

The error, PDI, and posterior uncertainty weightings of the evaluator function (eqn (6)) were set to *w*_1_ = 0.5, *w*_2_ = 0.2 and *w*_3_ = 2, respectively. These weightings were chosen to reflect an uncertainty-first selection strategy for an initial inverse design framework operating from a limited dataset. The posterior uncertainty weighting was assigned the highest value to prioritise candidates for which the surrogate model has the greatest confidence. The size error weighting was assigned an intermediate value to prioritise candidates whose predicted size most closely matches the target size. The PDI weighting was assigned the lowest value to prevent over-constraining candidate selection to a single nearest neighbour with the lowest PDI value, which would reduce the diversity of the candidate conditions and risk overfitting the selection to noisy PDI estimates. The absence of a systematic sensitivity analysis over the weightings is acknowledged as a limitation of the current framework.

The results for the inverse design predictions for both reactor configurations are presented in Fig. 7(a) and (b). The average error of the batch reactor predictions in the quartiles Q1, 2, 3, and 4 was 6.1, 1.7, 3.0, and 2.4%, respectively, with an average size error of 3.4%. For the flow reactor the average error was 18.6, 9.7, 2.5, and 3.5% in Q1, 2, 3, and 4, respectively, with an overall average error of 7.4%. Across both platforms, the elevated uncertainty weighting in eqn (6) ensured that selected candidates were drawn from well-sampled regions of the parameter space, which contributed to the overall robustness of the predictions. Dispersity outcomes further support the framework's effectiveness, with 8 out of 10 flow reactor predictions and 6 out of 11 batch reactor predictions achieving average PDI < 0.15, confirming that the *k*NN-based PDI constraint meaningfully biased the candidate selection toward monodisperse outcomes.

**Fig. 7:**
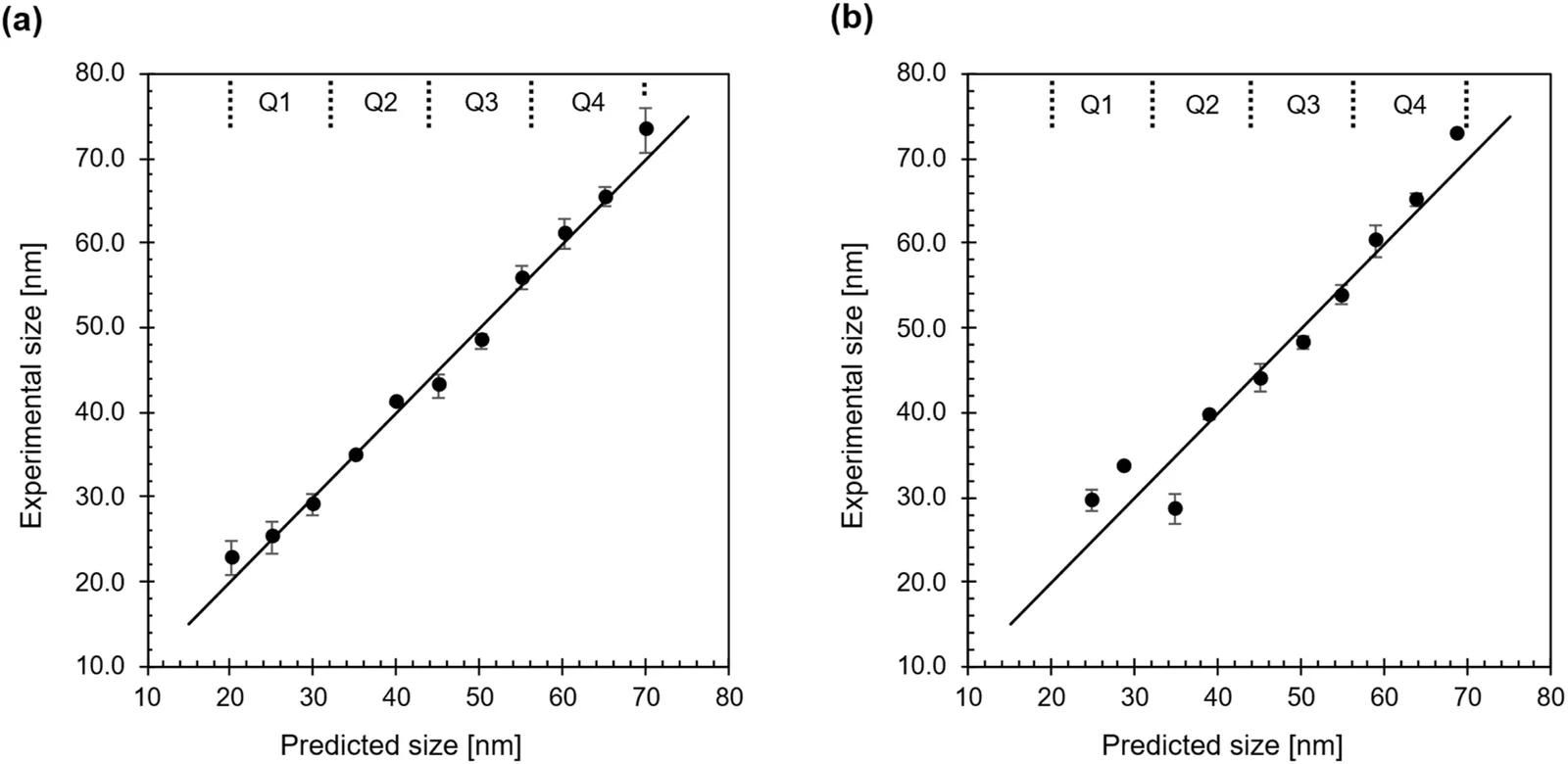
Optimisation results for Ag-NP prediction using the inverse-design framework for (a) batch and (b) flow synthesis.

The highest prediction errors occurred within Q1 of the flow reactor, yielding errors of 20.1, 17.1 and 17.6% for target sizes of 25, 29, and 35 nm, respectively, despite sufficient training data coverage within this quartile. This degradation is likely attributable to mixing limitations under conditions where the reduction of AgNO_3_ by SC and TA occurs on a timescale comparable to that of mixing. In this work, reagents were assumed to be homogeneous prior to the induction period. However, this assumption may not hold under rapid reduction conditions, as Bastús *et al.* (2014) reported reaction times of less than 30 s under low [TA]_rxn_ and high [SC]_rxn_ conditions.^11^ Cabeza *et al.* (2012) further demonstrated that for the synthesis of gold NPs from tetrachloroauric acid *via* rapid reduction by sodium borohydride, particle size and PSD were governed by internal mixing within the slugs in segmented flow synthesis.^44^ Consequently, optimisation of flow rate, slug length, and segmenting fluid presents a practical approach to controlling mixing and particle size under analogous conditions in Ag-NP synthesis. The incorporation of such hydrodynamic parameters into future model iterations would enable explicit accounting for platform-specific mixing effects, enhancing the model's ability to capture hydrodynamic influences on particle size and dispersity.

Further, for target sizes within Q4 for the flow reactor, the framework selected conditions characterised by elevated temperature (>100 °C) and high [TA]_rxn_, which experimentally yielded particles with elevated dispersity (PDI > 0.15). Alternatively, conditions with elevated temperature at low [SC]_rxn_ would have produced NPs of comparable size with lower dispersity but were not chosen by the model due to its reduced predictive accuracy in this sparsely sampled region. By optimising the evaluator function weightings and by incorporating active learning, whereby new experiments are added to the training set, these limitations can be addressed by improving surrogate fidelity.

The predictive results also exhibited precise, reproducible control over the Ag-NP hydrodynamic diameter. Triplicate syntheses were performed for each target size, with the mean and standard deviation for each shown in Fig. 7. The mean relative standard deviation across all batch and flow reactor predictions was 3.5% and 2.3%, respectively, consistent with the experimental precision of DLS measurements. The largest uncertainty across the results were observed for the batch reactor within Q1, with mean relative standard deviations of 9.0% and 7.7% for the 20 and 25 nm target sizes, respectively. This increased variability can be attributed to the rapid nucleation which occurs at such low particle sizes, increasing the sensitivity of the final particle size to local concentration gradients and mixing homogeneity at the point of reagent addition, both of which are inherently difficult to control in the batch reactor.

As an initial surrogate-based inverse design framework, several limitations remain. The *k*NN-based PDI estimation is inherently constrained by the coverage of the training dataset, potentially limiting exploration of the synthesis space beyond the training distribution. This is compounded by the unstructured nature of the training dataset, which inherently does not provide uniform coverage of the synthesis space. Further, PDI is estimated from a single neighbour, therefore any measurement noise in that neighbour's PDI value is directly inherited by the candidate without averaging or correction, potentially yielding unreliable PDI scores in noisy regions of the dataset. This effect is apparent in the triplicate synthesis results, where variability in the PDI across the repeats was significant at smaller particle sizes, particularly in Q1. This can also explain the poorer predictive performance of the flow reactor in this region, where the scoring function selected candidates with neighbours with low PDI but higher size error. This suggests region-specific weightings of the scoring function could better address the distinct challenges within each quadrant of the dataset. For example, reducing the target size range when selecting neighbouring candidates and increasing the value of the error weighting (*w*_1_) for Q1 predictions would improve the predictions of the flow reactor in this region.

Above 60 nm hydrodynamic diameter, nanoparticle morphology becomes an increasingly confounding factor, as non-spherical and faceted morphologies influence the hydrodynamic diameter. By incorporating UV-vis derived descriptors such as LSPR peak position and the full width at half maximum (FWHM) values into the inverse design framework could help to address this limitation. The LSPR peak provides an independent estimate of mean particle size, while the FWHM is a more robust indicator of dispersity than PDI, as demonstrated by the consistency of FWHM values across triplicate syntheses in this work, providing a route to more reliable dispersity estimation without dependence on a single *k*NN neighbour.^45^ Integration of these descriptors into the composite scoring function could enable more robust multi-objective optimisation beyond hydrodynamic diameter alone, potentially extending control to morphology and dispersity simultaneously.

## 4 Conclusions

Precise synthesis of Ag-NPs of controlled size is critical for application-specific performance, yet identification of optimal synthesis conditions remains reliant on trial-and-error approaches. Here, Ag-NPs were synthesised in both batch and flow reactors *via* citrate- and tannic acid-mediated reduction of silver nitrate, generating a high-quality dataset spanning 119 conditions. Five regression algorithms were subsequently evaluated as candidate surrogate models, with the GP regression model demonstrating superior predictive performance (CV score = 0.80). A two-stage inverse design framework was then implemented, in which random sampling of the parameter space identified conditions capable of achieving user-defined target Ag-NP hydrodynamic diameters. The candidate conditions were ranked *via* a PDI-informed scoring function to minimise prediction uncertainty and nanoparticle dispersity. The model was validated across 21 predictions, achieving average size errors of 3.4% and 7.4% for the batch and flow reactors, respectively. In summary, the surrogate -based inverse design framework developed in this study enables accurate prediction of synthesis conditions for target Ag-NP hydrodynamic diameters using limited experimental data, reducing reliance on trial-and-error optimisation. This approach has the potential to accelerate nanomaterials development, improve resource efficiency and support the adoption of ML-driven materials design workflows.

## Author contributions

Joseph Carver: writing – review and editing, writing – original draft, Visualisation, validation, resources, project administration, methodology, investigation, funding acquisition, formal analysis, data curation, Conceptualisation. YM John Chew: writing – review and editing, validation, supervision, resources, investigation, funding acquisition, formal analysis. Semali Perera: writing – review and editing, validation, supervision, resources, investigation, funding acquisition, formal analysis. Sourav Chatterjee: writing – review and editing, validation, supervision, resources, investigation, funding acquisition, formal analysis.

## Conflicts of interest

The authors declare that they have no known competing financial interests or personal relationships that could have appeared to influence the work reported in this paper.

## Supplementary Material

NA-008-D6NA00557H-s001

## Data Availability

The code and training data for this article can be found at https://github.com/josephwcarver1-bot/Data-driven-machine-learning-optimisation-of-silver-nanoparticle-synthesis/releases/tag/v1.0.0 with DOI—https://doi.org/10.5281/zenodo.20701488. The version of the code employed for this study is version 1.0.0. Supplementary information (SI): (1) TEM images; (2) hyperparameter tuning; (3) inverse design framework; (4) optimisation results. See DOI: https://doi.org/10.1039/d6na00557h.

## References

[cit1] Sati A., Ranade T. N., Mali S. N., Ahmad Yasin H. K., Pratap A. (2025). ACS Omega.

[cit2] Kenmotsu S., Hirasawa M., Tamadate T., Matsumoto C., Osone S., Inomata Y., Seto T. (2024). ACS Omega.

[cit3] Shamaeizadeh A., Razi Astaraei F., Kasaeian A. (2024). Results Opt..

[cit4] Revathy R., Joseph J., Augustine C., Sajini T., Mathew B. (2022). Environ. Sci.: Adv..

[cit5] Bouafia A., Laouini S. E., Ahmed A. S. A., Soldatov A. v., Algarni H., Feng Chong K., Ali G. A. M. (2021). Nanomaterials.

[cit6] Gaddam S. A., Kotakadi V. S., Allagadda R., Vasavi T., Velakanti S. G., Samanchi S., Thangellamudi D., Masarapu H., Maheswari P U., Ch A. R., Zereffa E. A. (2024). Sci. Rep..

[cit7] Abbas R., Luo J., Qi X., Naz A., Khan I. A., Liu H., Yu S., Wei J. (2024). Nanomaterials.

[cit8] Yaqoob A. A., Umar K., Ibrahim M. N. M. (2020). Appl. Nanosci..

[cit9] Zhang Q., Li N., Goebl J., Lu Z., Yin Y. (2011). J. Am. Chem. Soc..

[cit10] Heydaryan K. (2026). J. Sol-Gel Sci. Technol..

[cit11] Bastús N. G., Merkoçi F., Piella J., Puntes V. (2014). Chem. Mater..

[cit12] Pinheiro L. D. S. M., Sangoi G. G., Vizzotto B. S., Moreno Ruiz Y. P., Galembeck A., Pavoski G., Espinosa D. C. R., Machado A. K., da Silva W. L. (2024). Mater. Chem. Phys..

[cit13] Khatoon U. T., Velidandi A., Nageswara Rao G. V. S. (2023). Mater. Chem. Phys..

[cit14] Malassis L., Dreyfus R., Murphy R. J., Hough L. A., Donnio B., Murray C. B. (2016). RSC Adv..

[cit15] Agnihotri S., Mukherji S., Mukherji S. (2014). RSC Adv..

[cit16] Thanh N. T. K., Maclean N., Mahiddine S. (2014). Chem. Rev..

[cit17] Oliveira M. M., Ugarte D., Zanchet D., Zarbin A. J. G. (2005). J. Colloid Interface Sci..

[cit18] Martínez-Castañón G. A., Niño-Martínez N., Martínez-Gutierrez F., Martínez-Mendoza J. R., Ruiz F. (2008). J. Nanopart. Res..

[cit19] Yang L., Wang H., Leng D., Fang S., Yang Y., Du Y. (2024). Chem. Eng. J..

[cit20] Richard C., Pichon P.-A., Nováková J., Najafabadi N. I., Sá J. (2026). Discover Nano.

[cit21] Tao H., Wu T., Aldeghi M., Wu T. C., Aspuru-Guzik A., Kumacheva E. (2021). Nat. Rev. Mater..

[cit22] Nathanael K., Pico P., Kovalchuk N. M., Lavino A. D., Simmons M. J. H., Matar O. K. (2022). Chem. Eng. J..

[cit23] Prasad A., Santra T. S., Jayaganthan R. (2024). Metals.

[cit24] Nathanael K., Cheng S., Kovalchuk N. M., Arcucci R., Simmons M. J. H. (2023). Chem. Eng. Res. Des..

[cit25] Furxhi I., Faccani L., Zanoni I., Brigliadori A., Vespignani M., Costa A. L. (2024). Comput. Struct. Biotechnol. J..

[cit26] Kim H. J., Carbone M. R., Lu F., Qu X., Reyes K., Zhang H., Zhang L., Gang O., Zhang Y. (2026). J. Am. Chem. Soc..

[cit27] Vaddi K., Chiang H. T., Grey A., Wylie Z. R., Pozzo L. D. (2025). npj Comput. Mater..

[cit28] Yang G., Xiao Q., Zhang Z., Yu Z., Wang X., Lu Q. (2025). iScience.

[cit29] Ha C. S., Yao D., Xu Z., Liu C., Liu H., Elkins D., Kile M., Deshpande V., Kong Z., Bauchy M., Zheng X. (2023). Nat. Commun..

[cit30] Dai M., Jiang Y., Yang F., Chattoraj J., Xia Y., Xu X., Zhao W., Dao M. H., Liu Y. (2024). Neural Networks.

[cit31] Mahdian M., Ender F., Pardy T. (2026). Sci. Rep..

[cit32] Mekki-Berrada F., Ren Z., Huang T., Wong W. K., Zheng F., Xie J., Tian I. P. S., Jayavelu S., Mahfoud Z., Bash D., Hippalgaonkar K., Khan S., Buonassisi T., Li Q., Wang X. (2021). npj Comput. Mater..

[cit33] Shafaei A., Khayati G. R. (2020). Measurement.

[cit34] Nathanael K., Cheng S., Kovalchuk N. M., Arcucci R., Simmons M. J. H. (2025). Chem. Eng. Res. Des..

[cit35] Grunloh N. R., Lee H. K. H. (2025). Mathematics.

[cit36] Carver J., Chew Y. J., Perera S., Moon S., Chatterjee S. (2026). Food Bioprod. Process..

[cit37] Sandoe H. E., Watzky M. A., Diaz S. A. (2019). Int. J. Chem. Kinet..

[cit38] Modena M. M., Rühle B., Burg T. P., Wuttke S. (2019). Adv. Mater..

[cit39] Bentea L., Watzky M. A., Finke R. G. (2017). J. Phys. Chem. C.

[cit40] Cheng Y., Wang F., Fang C., Su J., Yang L. (2016). J. Alloys Compd..

[cit41] Ranoszek-Soliwoda K., Tomaszewska E., Socha E., Krzyczmonik P., Ignaczak A., Orlowski P., Krzyzowska M., Celichowski G., Grobelny J. (2017). J. Nanopart. Res..

[cit42] Kuznetsova E. v., Kuznetsov N. M., Kalinin K. T., Lebedev-Stepanov P. v., Novikov A. A., Chvalun S. N. (2022). Colloid J..

[cit43] Pourkamali-Anaraki F., Husseini J. F., Pineda E. J., Bednarcyk B. A., Stapleton S. E. (2024). Eng. Appl. Artif. Intell..

[cit44] Cabeza V. S., Kuhn S., Kulkarni A. A., Jensen K. F. (2012). Langmuir.

[cit45] Borah R., Verbruggen S. W. (2022). Colloids Surf., A.

