# Supplementary material for: Data-driven machine learning optimisation of silver nanoparticle synthesis
Source: Nanoscale Adv. 2026 Sep 21;8(21):6229–42. doi: 10.1039/d6na00557h (PMC13592209; doi:10.1039/d6na00557h)
Supplement: Supplementary file 1 [file NA-008-D6NA00557H-s001.pdf]

Supplementary Information (SI) for Nanoscale Advances.
This journal is © The Royal Society of Chemistry 2026

Supporting Information (SI)

Data-driven machine learning optimisation of silver nanoparticle synthesis

Joseph Carver[1*], YM John Chew[1], Semali Perera[1], Sourav Chatterjee[1*]

[1] *Department of Chemical Engineering, University of Bath, Bath, BA2 7AY, UK.*

Corresponding Author

*E-mail: Joseph Carver(jc2899@bath.ac.uk), Sourav Chatterjee (sc999@bath.ac.uk)

S.1 TEM Images

Fig. S1, S2 and S3 show additional TEM images for the Ag-NPs synthesised in the batch and flow reactors.

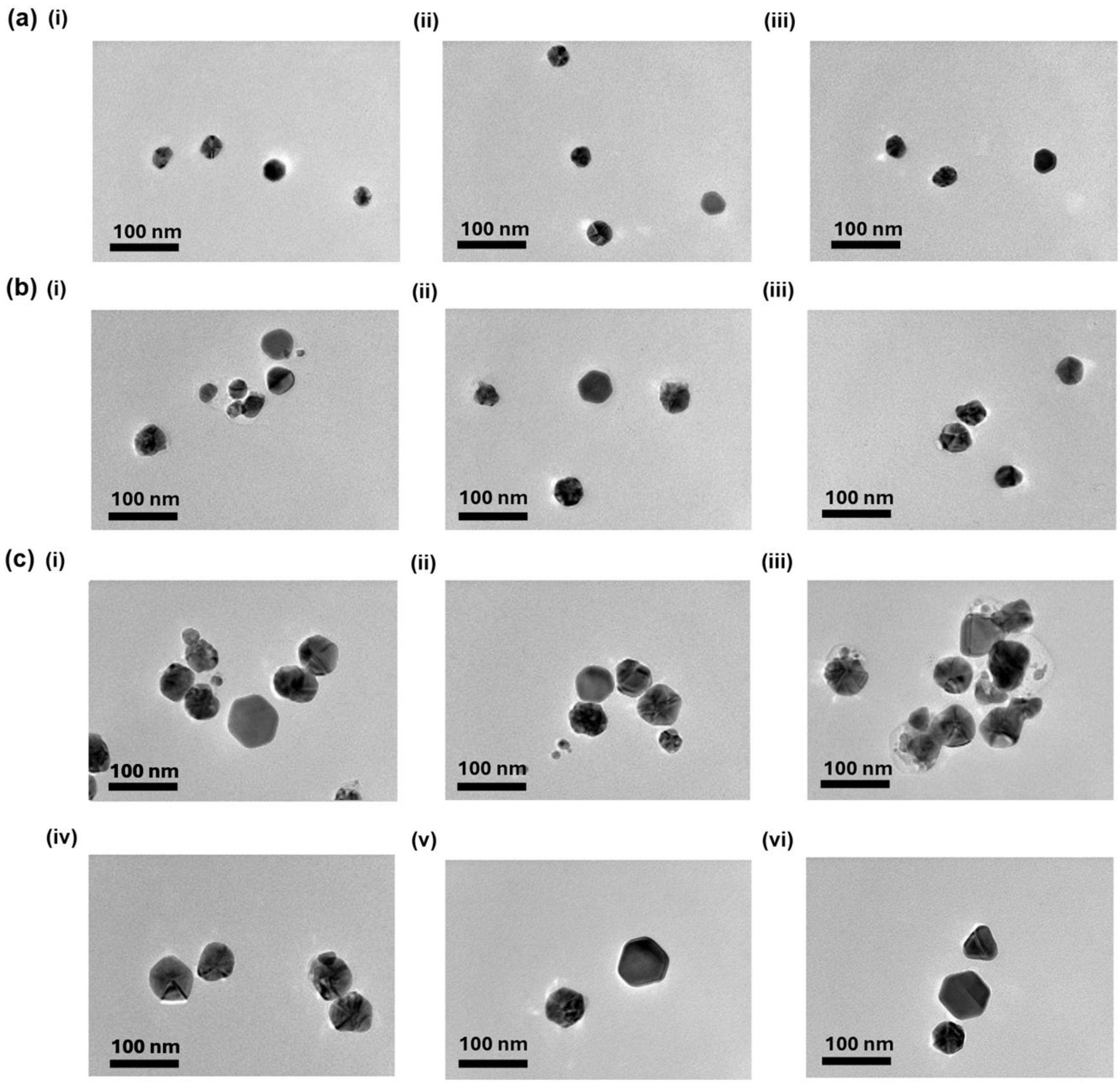

Fig. S1 TEM images for Ag-NPs synthesised in the batch reactor with hydrodynamic diameters of (a) 35 nm, (b) 44 nm, and (c) 57 nm.

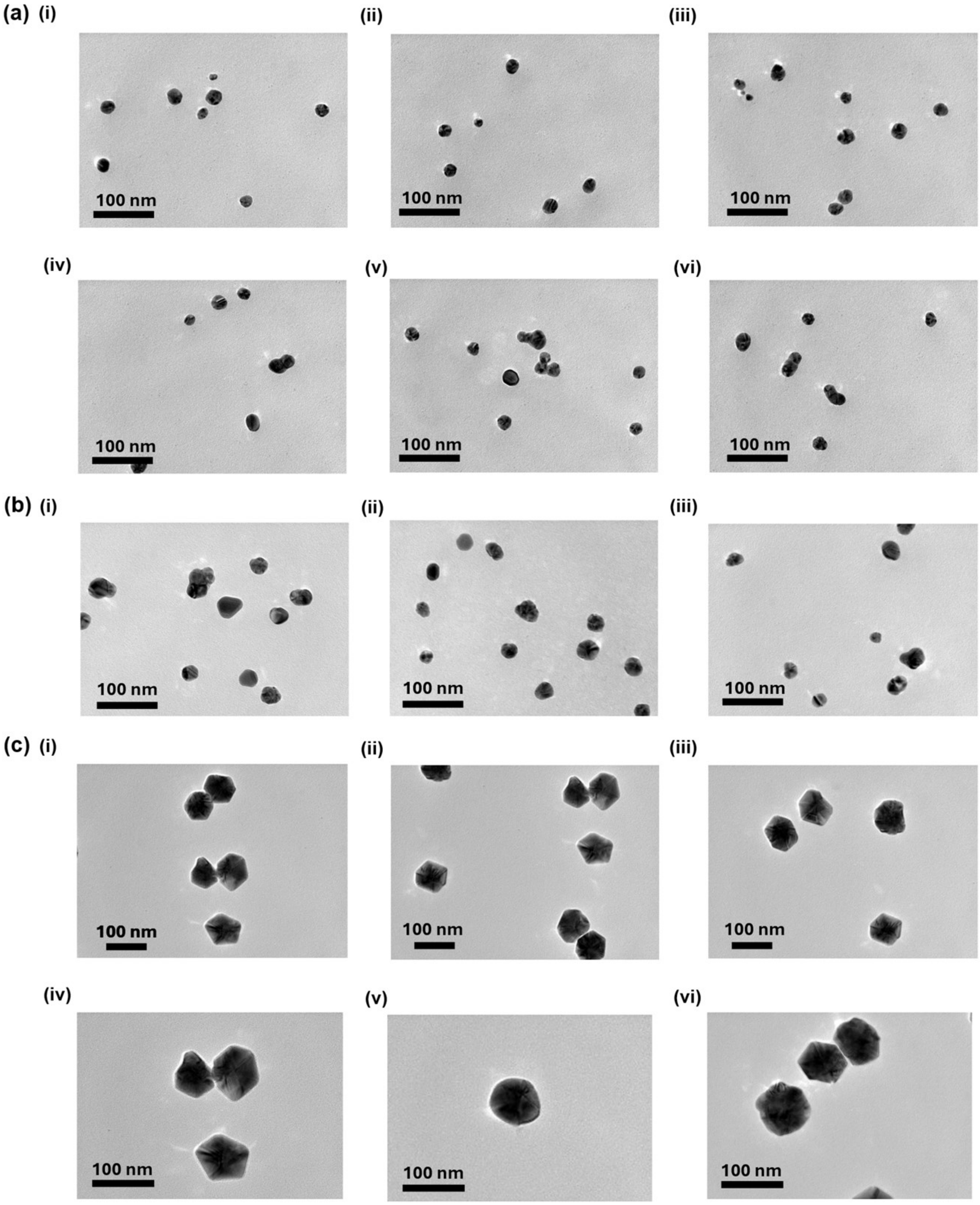

Fig. S2 TEM images for Ag-NPs synthesised in the flow reactor with hydrodynamic diameters of (a) 29 nm, (b) 37 nm, and (c) 67 nm.

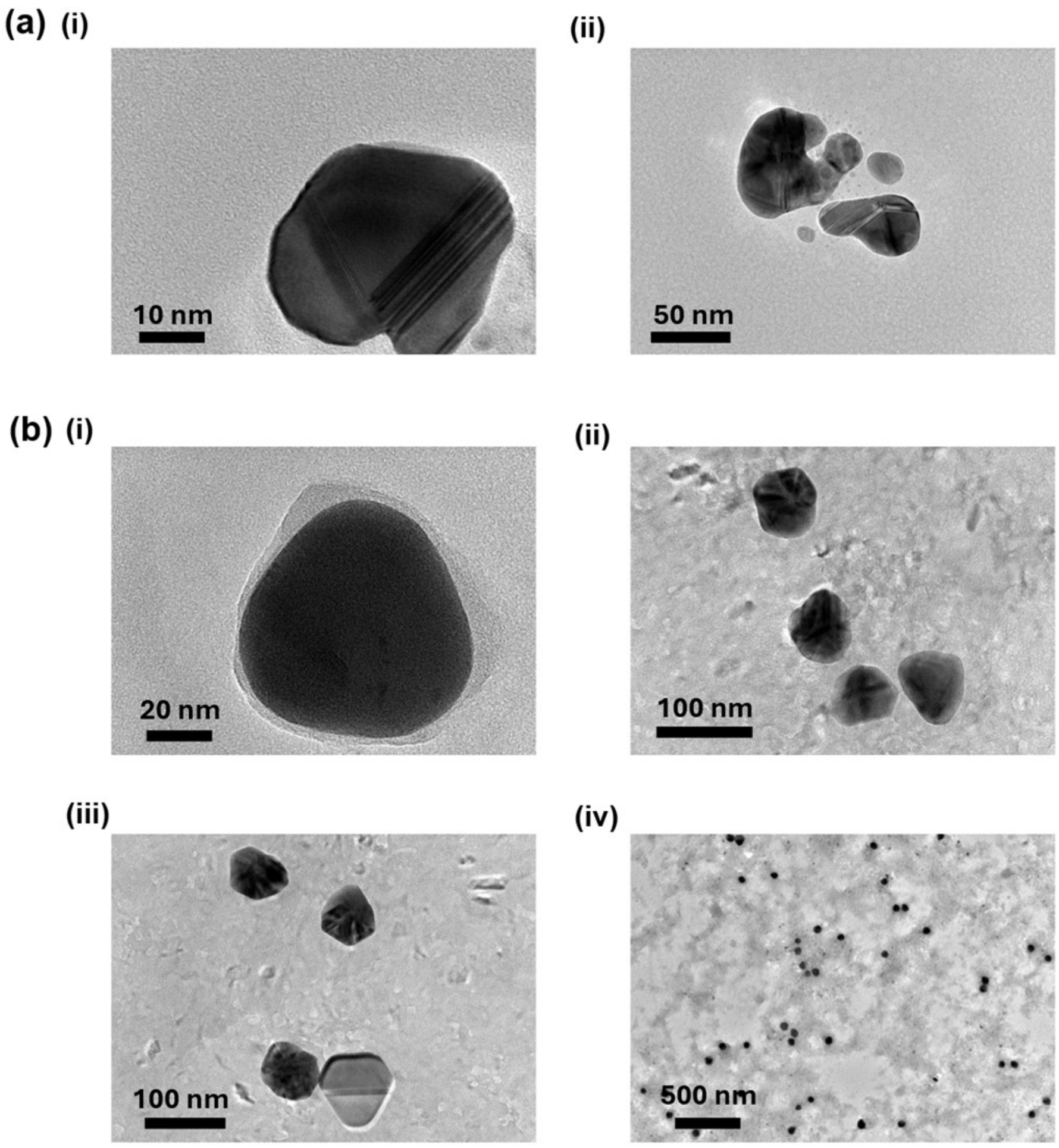

Fig. S3 TEM micrographs for Ag-NPs synthesised in the flow reactor under (a) high $[TA]_{rxn}$ and (b) low $[SC]_{rxn}$ with hydrodynamic diameters of 69.4 and 65.8 nm, respectively.

S.2 Hyperparameter Tuning

The training parameters for the three regression models studied are given in Table S1.

Table S1. Training parameters for the three regression models, random forest (RF), extreme gradient boosting (XGBoost), and Gaussian process (GP).

Model	Hyperparameter	Tested range/values	Best value
RF	n_estimators	[100, 200, 300]	200
	min_samples_split	[2, 3, 4, 5, 10]	2
	min_samples_leaf	[1, 2, 4, 6]	1
	max_features	['sqrt', 'log2']	sqrt
	max_depth	[None, 10, 20, 30]	None
	bootstrap	[True, False]	True
XGBoost	subsample	[0.7, 0.9, 1.0]	0.8
	reg_lambda	[1, 5, 10]	10
	reg_alpha	[0, 0.1, 1]	1
	n_estimators	[100, 200, 300]	150
	max_depth	[3, 4, 5, 6]	3
	learning_rate	[0.01, 0.05, 0.1, 0.2]	0.28
	gamma	[0, 0.1]	0.1
	colsample_bytree	[0.6, 0.8, 1]	0.6
GP	gpr_kernel	RBF: l ∈ [0.1, 0.316, 1, 3.16, 10]<br>Matérn (ν = 1.5): l ∈ [0.01, 0.1, 1, 10, 100]<br>Rational Quadratic: l ∈ [0.01, 1, 100], α ∈ [0.1, 1, 10]<br>ConstantKernel C ∈ [1e-3, 1e5] + WhiteKernel	Matérn (ν = 1.5): l = 0.1, C = 1, noise level = 1
	gpr_alpha	[1e-10, 1e-6, 1e-4, 1e-2]	0.01

Fig. S4 shows the list of feature importance for each algorithm after training.

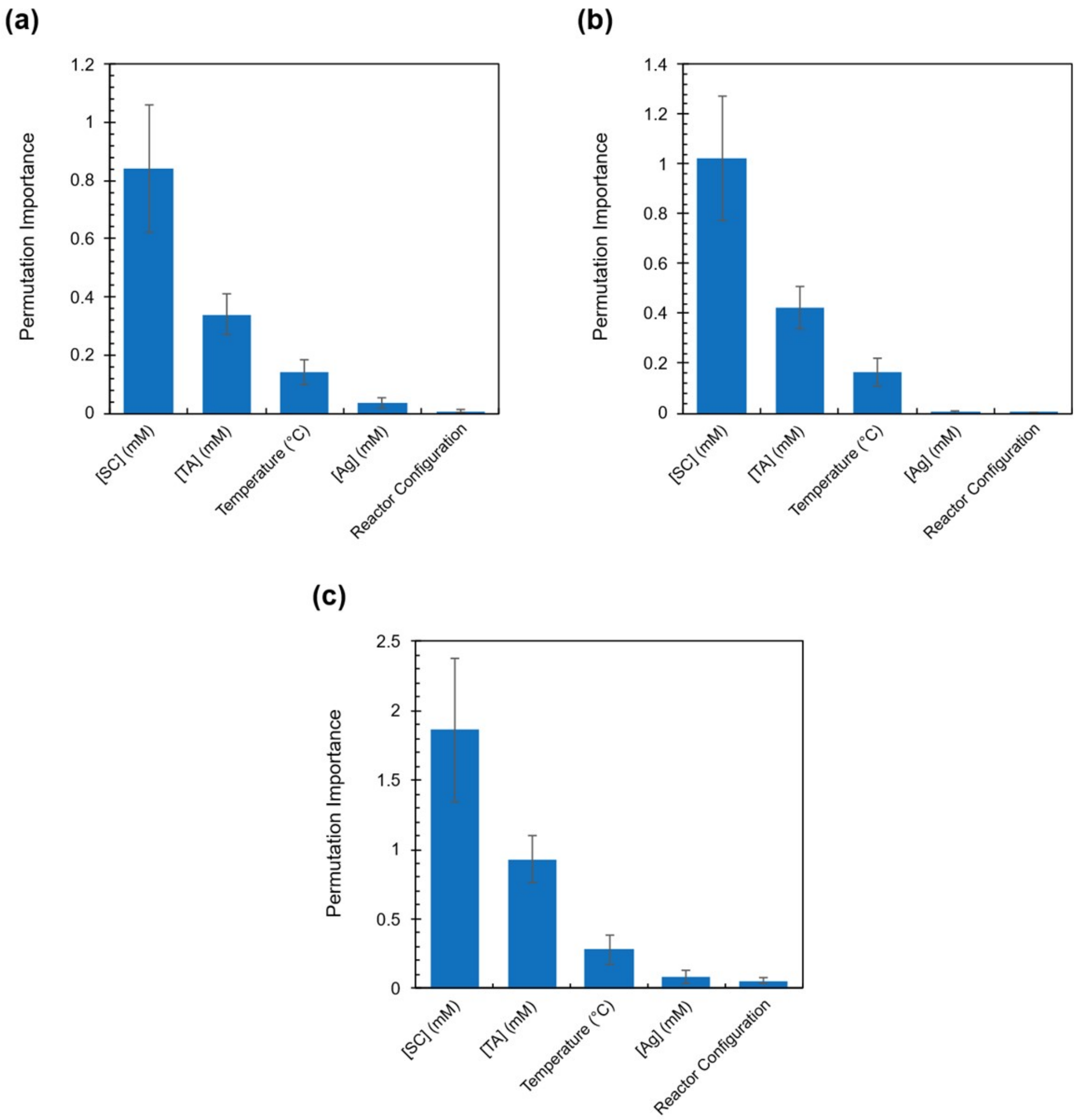

Fig. S4 Premutation feature importance of (a) RF, (b) XGBoost, and (c) GP models.

S.3 Inverse Design Framework

S3.1 Candidate Generation

Candidate synthesis conditions were generated via random sampling of the synthesis parameter space within the bounds defined in Table S2. A fixed random seed of 42 was chosen to ensure reproducibility. The reactor configuration was fixed as a categorical input (batch = 0, flow = 1) for each inverse design run.

Table S2. Synthesis parameter bounds used for uniform random sampling during candidate generation in the inverse design framework.

Feature	Min	Max
Temperature (°C)	60	125
Concentration of $AgNO_3$ in the reaction mixture = $[Ag]_{rxn}$ (mM)	0.1	0.50
Concentration of TA in the reaction mixture = $[TA]_{rxn}$ (mM)	0.01	0.62
Concentration of SC in the reaction mixture = $[SC]_{rxn}$ (mM)	0.10	3.96

S3.2 Implementation

The framework was implemented in Python using scikit-learn for GP regression and kNN estimation, NumPy for random sampling, and pandas for data handling.

S.4 Optimisation Results

The predictions and experimental results from the inverse modelling are presented in Table S3, S4 and S5.

Table S3. Optimised synthesis conditions predictions from the inverse modelling.

	Target size (nm)	Temp. (°C)	[Ag] (mM)	$[TA]_{rxn}$ (mM)	$[SC]_{rxn}$ (mM)
**Batch reactor**	20	88	0.48	0.14	1.75
	25	86	0.24	0.19	2.46
	30	79	0.26	0.50	2.29
	35	80	0.26	0.47	0.62
	40	82	0.50	0.47	0.41
	45	78	0.25	0.12	0.18
	50	83	0.48	1.69	0.53
	55	80	0.49	2.05	0.38
	60	90	0.49	2.28	0.27
	65	67	0.48	2.13	0.11
	70	101	0.46	2.31	0.11
**Flow reactor**	25	101	0.50	0.01	0.55
	29	86	0.49	0.02	0.71
	35	119	0.50	0.38	0.50
	39	102	0.49	0.05	0.48
	45	97	0.49	0.07	0.43
	50	91	0.49	0.07	0.30
	55	107	0.50	0.11	0.45
	59	117	0.49	0.13	0.48
	64	116	0.50	0.10	0.31
	69	115	0.49	0.17	0.43

Table S4. Prediction results of the batch inverse modelling.

Target (nm)	Model's prediction (nm)	Repeat No.	Size (nm)	Error (nm)	PDI (-)	LSPR peak (nm)	FWHM (nm)
20.0	20.0	1	22.0	2.0	0.214	401	65.2
		2	25.7	5.7	0.137	404	68.5
		3	20.9	0.9	0.214	399	57.2
25.0	25.0	1	26.3	1.3	0.136	404	72.6
		2	26.9	1.9	0.158	407	75.1
		3	22.5	2.5	0.238	406	65.0
30.0	30.0	1	28.8	1.2	0.220	409	-
		2	27.7	2.3	0.119	409	88.3
		3	30.6	0.6	0.081	409	84.0
35.0	35.0	1	35.0	0.0	0.081	420	79.4
		2	35.1	0.1	0.093	420	86.6
		3	34.5	0.5	0.102	417	80.1
40.0	39.9	1	40.9	0.9	0.097	424	70.3
		2	41.3	1.3	0.068	426	73.9
		3	41.2	1.2	0.093	427	74.0
45.0	45.0	1	44.3	0.7	0.102	427	73.5
		2	41.2	3.8	0.072	426	76.2
		3	44.0	1.0	0.095	426	74.7
50.0	50.0	1	48.2	1.8	0.117	430	77.0
		2	47.3	2.7	0.110	430	79.0
		3	49.6	0.4	0.196	429	76.1
55.0	55.1	1	56.7	1.7	0.150	442	94.3
		2	53.9	1.1	0.112	437	83.8
		3	57.1	2.1	0.232	438	84.8
60.0	60.1	1	63.5	3.5	0.209	454	121.0
		2	60.5	0.5	0.130	443	97.9
		3	59.1	0.9	0.122	445	101.5
65.0	65.0	1	67.0	2.0	0.193	474	151.3
		2	64.1	0.9	0.221	480	158.9
		3	65.2	0.2	0.230	475	140.3
70.0	69.8	1	72.9	2.9	0.197	485	154.7
		2	70.2	0.2	0.193	465	129.6
		3	76.9	6.9	0.150	471	143.7

Table S5. Prediction results of the flow inverse modelling.

Target (nm)	Model's prediction (nm)	Repeat No.	Size (nm)	Error (nm)	PDI (-)	LSPR peak (nm)	FWHM (nm)
25.0	24.7	1	28.6	3.6	0.084	408	-
		2	29.1	4.1	0.097	408	70.5
		3	31.3	6.3	0.246	411	75.8
29.0	28.8	1	33.4	4.4	0.159	416	80.5
		2	33.6	4.6	0.168	415	79.9
		3	33.9	4.9	0.149	412	76.3
35.0	34.9	1	31.1	3.9	0.077	414	73.1
		2	28.2	6.8	0.075	412	73.8
		3	26.9	8.1	0.015	410	71.2
39.0	38.9	1	40.2	1.2	0.144	420	70.1
		2	39.3	0.3	0.103	422	69.2
		3	39.3	0.3	0.133	421	69.5
45.0	45.1	1	46.3	1.3	0.157	426	76.6
		2	42.8	2.2	0.083	424	74.0
		3	43.2	1.8	0.100	424	73.6
50.0	50.1	1	47.4	2.6	0.121	430	76.4
		2	48.3	1.7	0.133	429	75.7
		3	49.0	1.0	0.108	427	77.7
55.0	54.8	1	52.5	2.5	0.136	430	86.6
		2	54.3	0.7	0.145	429	86.7
		3	55.0	0.0	0.165	430	89.3
59.0	59.0	1	60.1	1.1	0.137	441	106.2
		2	62.5	3.5	0.148	438	103.8
		3	58.1	0.9	0.151	441	111.8
64.0	63.9	1	66.2	2.2	0.147	449	120.1
		2	64.2	0.2	0.143	446	109.7
		3	65.3	1.3	0.168	448	115.4
69.0	68.7	1	73.0	4.0	0.163	462	155.1
		2	73.1	4.1	0.154	454	141.9
